# Neuroprotective effects of rutin, sodium selenite, and rutin-conjugated selenium nanoparticles in a social isolation model

**DOI:** 10.3389/fphar.2026.1782734

**Published:** 2026-04-15

**Authors:** Khaled M. Alam-ElDein, Abanoub A. S. Shaker, Manal F. El-Khadragy, Ibrahim K. M. Alabbadi, Ahmed E. Abdel Moneim, Alzahraa A. Elhemiely, Nievin Ahmed Mahran, Triveena M. Ramsis, Shaimaa H. El-Fayoumi, Mohamed A. Ali, Eman Fayad, Haitham Ibrahim El-Mekkawy, Mohamed H. A. Gadelmawla

**Affiliations:** 1 Molecular Biology and Biotechnology Department, School of Biotechnology, Badr University in Cairo, Badr City, Egypt; 2 Department of Biology, College of Science, Princess Nourah bint Abdulrahman University, Riyadh, Saudi Arabia; 3 Medical Services Administration, Taif University, Taif, Saudi Arabia; 4 Unit of Scientific Research, Applied College, Qassim University, Buraydah, Saudi Arabia; 5 Department of Pharmacology, Egyptian Drug Authority (EDA)-Formerly NODCAR, Giza, Egypt; 6 Department of Biochemistry, Faculty of Biotechnology, Sinai University, Ismailia, Egypt; 7 Department of Pharmaceutical Chemistry, Faculty of Pharmacy, Sinai University, Ismailia, Egypt; 8 Department of Pharmacology and Toxicology, Faculty of Pharmacy, Heliopolis University, Cairo, Egypt; 9 School of Biotechnology, Badr University in Cairo, Badr City, Egypt; 10 Department of Biotechnology, College of Sciences, Taif University, Taif, Saudi Arabia; 11 Department of Biology, College of Science, King Khalid University, Asir, Saudi Arabia; 12 Department of Life Sciences, Faculty of Biotechnology, Sinai University, Ismailia, Egypt

**Keywords:** nanoparticles, neuroinflammation, oxidative stress, rutin, selenium, social isolation

## Abstract

**Background:**

Social isolation (SI) is a long-standing experimental paradigm that models schizophrenia-like behavioural and neurobiological changes caused by the action of oxidative stress, neuroinflammation, apoptosis, and neurotransmitter imbalance. The scope of this paper was to comparatively assess the effectiveness of rutin, sodium selenite (Na_2_SeO_3_), and rutin-conjugated selenium nanoparticles (RUT-SeNPs) to determine their neuroprotective activity using a rat model of neurobehavioral impairment following SI.

**Methods:**

In silico analysis of Rutin activity including *in silico* ADMET and toxicity, and molecular docking. Forty-two healthy male albino rats were allocated equally in to six groups; Control group, Social isolation group (SI), SI treated with Olanzapine (SI&OLA), SI treated with Rutin group (SI&RUT), SI treated with the sodium selenite group (SI&Na_2_SeO_3_), and SI treated with Se nanoparticles biosynthesized using the Rutin group (SI&RUT-SeNPs). All groups undergone biochemical analysis including behavioral tests, assessment of neural function, oxidative stress, inflammation, and apoptosis, histopathological analysis, immunohistochemistry, and gene expression.

**Results:**

SI induction produced significant behavioral, biochemical, neurochemical, and structural impairments compared with controls (*P* < 0.05). SI rats showed marked reductions in locomotion, sucrose preference, social interaction, antioxidant capacity (Nrf2, SOD, CAT, GSH), neurotransmitters (serotonin, dopamine, GABA, glycine), and BDNF, alongside significant increases in oxidative stress markers (8-OHdG, MDA, NO), inflammatory cytokines (TNF-α, IL-1β, NF-κB), apoptotic mediators (Bax and Caspase-3), GFAP, and histological degeneration (*P* < 0.05). Across all assessments, RUT-SeNPs produced the strongest and statistically significant recovery, yielding values comparable to control and significantly superior to SI, SI&OLA, and SI&RUT groups (*P* < 0.05). RUT-SeNPs normalized behavioral outcomes, antioxidant status, cytokine levels, apoptotic markers, neurotransmitters, BDNF/GFAP balance, and cortical histoarchitecture.

**Conclusion:**

Rutin and sodium selenite provided significantly protective effects across behavioral, biochemical, and neurochemical parameters. Among all treatments, RUT-SeNPs produced markedly attenuated, restoring nearly all measured markers including redox balance, cytokines, apoptosis regulators, neurotransmitters, BDNF/GFAP levels, and cortical histology to values comparable to controls.

## 1 Introduction

Schizophrenia (SCH) is a complicated and devastating mental condition that often begins in late adolescence or early maturity. It is defined by complex interplay between genetic, neural development, and environmental variables that disrupt normal brain circuitry and neurochemical homeostasis (Solmi et al., 2023). Affecting nearly 1% of the population, schizophrenia remains a significant health challenge, contributing to profound functional impairment, socioeconomic burden, and reduced life expectancy (Hadar et al., 2020). Beyond its psychiatric manifestations, the disorder exerts systemic effects, predisposing patients to metabolic, cardiovascular, and endocrine abnormalities that amplify morbidity and mortality (He et al., 2021; Popa et al., 2025). Cases with SCH suffer social isolation (SI), which is both self-imposed and a social reaction to their symptoms and functional disability (Powell and Swerdlow, 2023). SI was potentially causally associated with an increased risk of schizophrenia. A large cohort and Mendelian randomization study found that socially isolated individuals had significantly higher schizophrenia risk than non-isolated groups (Wu et al., 2025). Cases with schizophrenia are more socially isolated and have reduced social motivation compared to healthy individuals (Green et al., 2024).

At the molecular level, SI is linked with multifaceted neurobiological disturbances. Disturbances in dopaminergic signaling, especially mesolimbic hyperactivity and mesocortical hypofunction, underlies the hallmark positive and negative symptoms (Drevinskaite et al., 2025). However, contemporary research highlights broader neurotransmitter imbalances involving glutamatergic, GABAergic, and cholinergic systems, which collectively impair cognition, emotion regulation, and sensory processing (Ma et al., 2022; Bojesen et al., 2025). Concurrently, oxidative stress (OS) and neuroinflammation have participated as pivotal pathologic mechanisms (Cui et al., 2024). Elevated levels of malondialdehyde (MDA), nitric oxide (NO), and TNF-α and IL-1β coincide with reduced antioxidant defenses, including superoxide dismutase (SOD), catalase (CAT), and glutathione (GSH) (Ashour et al., 2025; Craven et al., 2025). Impaired activation of the Nrf2 pathway further disrupts redox balance, causing mitochondrial dysfunction and enhanced neuronal apoptosis via caspase-3 and Bax/BCL-2 dysregulation (Bojesen et al., 2025; Mohib et al., 2025; Mansour et al., 2026).

The use of social isolation rearing is well-known as a neurodevelopmental experimental model that can recreate the phenotypes of the schizophrenia disease, specifically schizophrenic negative-like symptoms. These are social withdrawal, lack of motivation drive, anhedonia as well as exploratory behavior. Although some behavioral tests used in social isolation models might also intersect with stress-/depression-related paradigms, the current study would be understood through a schizophrenia-like lens and a negative symptom domain together with the neurobiological dysfunctions (Bove et al., 2022; Karim et al., 2022; Mansour et al., 2025).

In this context, natural bioactive compounds with antioxidants and anti-inflammatory capacities offer promising neurotherapeutic potential. Rutin, a plant-derived flavanol glycoside, has demonstrated robust neuroprotective actions via upregulation of the Nrf2/HO-1 axis, downregulation of NF-κB signaling, restoration of monoaminergic neurotransmission, and attenuation of neuroinflammation (Ganeshpurkar and Saluja, 2017; Onaolapo et al., 2021).

Rutin’s possesses potential treatment for neurodegenerative diseases and cognitive impairments. Among the most relevant mechanisms implicated are the effect on change of the oxidant-antioxidant balance linked with neuronal cell death; and reducing the inflammatory component of neurodegeneration. Rutin’s physicochemical properties, which affect metal chelation and bioavailability, are also explored (Khan et al., 2009; Javed et al., 2012; Habtemariam, 2016).

Selenium, a vital trace element, performs an important function in preserving redox balance and neuronal health as a cofactor of antioxidant selenoenzymes including GSH peroxidase (Ansari et al., 2024). Selenium deficiency is linked to mood disturbances, cognitive dysfunction, and heightened susceptibility to oxidative injury. Recent advances have highlighted selenium nanoparticles (SeNPs) as a superior therapeutic form, offering improved bioavailability, cellular uptake, and reduced toxicity compared to conventional selenium compounds (Zhu et al., 2023). SeNPs possess powerful anti-inflammatory impact in neurodegenerative and stress-induced brain injury models. When synthesized through green chemistry approaches using rutin as both a reducing and capping agent (Al Omairi et al., 2022), the resulting rutin-conjugated selenium nanoparticles (RUT-SeNPs) combine the neuroprotective potential of both agents, yielding enhanced free radical scavenging capacity, stabilization of antioxidant defenses, and attenuation of neuronal apoptosis (Zaghloul et al., 2022).

Pharmacologically, olanzapine, a second-generation antipsychotic, remains a cornerstone in schizophrenia management due to its broad receptor antagonism across dopaminergic (D_2_) and serotonergic (5-HT_2_A/2C) systems, as well as activity on histaminergic, muscarinic, and adrenergic receptors (Wesner, 2024). Despite its robust efficacy against both positive and negative symptoms, long-term olanzapine therapy is limited by metabolic side effects, motivating the exploration of safer, natural, or nanoformulated alternatives (Taipale et al., 2025).

Although both rutin and selenium have independently demonstrated neuroprotective properties, their therapeutic efficacy may be limited by suboptimal bioavailability, metabolic instability, and restricted cellular uptake. Nanoparticle-based delivery systems have been proposed as a strategy to overcome these limitations by improving physicochemical stability, facilitating cellular internalization, and enabling synergistic redox modulation. Accordingly, the present study was designed as a comparative evaluation, incorporating free rutin, ionic selenium, and rutin-conjugated selenium nanoparticles within the same SI experimental framework. This design allows assessment of whether nano-formulation confers functional advantages over conventional formulations under identical biological conditions, without presupposing nano-specific mechanisms.

## 2 Materials and methods

### 2.1 In silico ADMET

ProTox-3.0 (https://tox.charite.de/protox3/, accessed on 20 October 2025) and ADMETLab 3.0 (https://admetlab3.scbdd.com/, accessed on 20 October 2025) online tool was used to analyze Rutin (Elseedy et al., 2024; Saad et al., 2024; Fayed et al., 2025). Rutin SMILES was uploaded to the ADMETLab 3.0 and ProTox3.0 websites, which assessed its physicochemical characteristics, pharmacokinetic characteristics, ADME parameters, and toxicity profile (Ramsis et al., 2018; Ebrahim et al., 2025).

### 2.2 Docking

#### 2.2.1 Protein preparation

The 3D structure of 5-HT_2A_ receptor (5-HT_2A_R) (PDB: 8ZMG), human dopamine D3 receptor (PDB ID: 3PBL), Human iNOS (PDB ID: 3HR4), human 8-oxoguanine DNA Glycosylase (OGG1) (PDB ID: 6RLW), and Keap1 (PDB ID: 6ZEZ) was extracted from protein data bank (https://www.rcsb.org/) and prepared employing UCSF Chimera (Xia et al., 2009; Chien et al., 2010; Chen et al., 2015; Visnes et al., 2020; Pallesen et al., 2021; Oguma et al., 2024). The protein was created by eliminating water molecules, incorporating hydrogen atoms and charges, and minimizing energy. The resulting protein was introduced in AutoDock. Hydrogens were added, Kollman charges and AD4 atom parameters were assigned.

#### 2.2.2 Ligand preparation

Avogadro software was used to create the rutin structure and refine its geometry under MMFF94 forcefield (Hanwell et al., 2012). After the ligand was loaded into Autodock, Gasteiger charges were assigned and hydrogens were added (Elseedy et al., 2024).

#### 2.2.3 Grid parameters

The bound ligand orientation was used to produce the grid, which had a resolution of 0.375 Å. Table 1 contains the grid box coordinates.

**TABLE 1:** Grid box dimensions.

PDB ID	Center coordinates			Size coordinates		
	*x*	*y*	*Z*	*x*	*y*	*z*
8ZMG	−20.5	13.45	37.16	30	30	35
3PBL	−0.9	−14.75	10.0	40	40	40
3HR4	1.3	9.4	−64.45	40	40	40
6RLW	−19.5	12.5	38.3	40	40	40
6ZEZ	38.5	23.5	−7.1	35	35	35

### 2.3 Molecular docking simulation

The search parameter for the Genetic Algorithm was used to initiate docking. Docked conformations were produced in descending order according to their docking energy using the Lamarckian genetic algorithm (Morris et al., 1998; Mai et al., 2003; Ali et al., 2007). Biovia Discovery Studio was used to display the data. Validation was enabled by repeating the identical procedures for the co-crystallized ligand. The DockRMSD online tool was used to determine the redocked pose RMSD value (Bell and Zhang, 2019; Ashour et al., 2025). The molecular docking analyses were performed to explore potential ligand–target interactions and to provide computational insight supportive of the *in vivo* findings; these simulations were not intended to serve as experimental confirmation of direct molecular binding or target inhibition.

### 2.4 Drugs

Rutin (CAS No. 153-18–4) was obtained from Sigma-Aldrich (St. Louis, MO, USA) and utilized without additional purification. The green synthesis of rutin-conjugated selenium nanoparticles (RUT-SeNPs) was accomplished via a simple aqueous reduction approach. Briefly, 10 mL of sodium selenite solution (Na_2_SeO_3_, 10 mM) was mixed with an equal volume of rutin solution (3.5 mg/mL) under continuous magnetic stirring at ambient temperature for 12 h. During the reaction, the color of the mixture gradually transitioned to a deep reddish hue, confirming the synthesis of SeNPs through the reduction of selenite ions and concurrent surface capping by rutin molecules. The resulting colloidal suspension was subjected to lyophilization using a vacuum freeze-drying system (FreeZone 4.5 L, Labconco, Marshall Scientific, Hampton, NH, USA) to yield a fine, dry RUT-SeNPs powder, which was subsequently stored in airtight vials and used for all downstream characterization and biological investigations.

### 2.5 Characterization of nanoparticles

#### 2.5.1 Zeta potential and particles size analysis

A Zetasizer Nano ZS90 (Malvern Panalytical Ltd., UK) was used in Helwan University to measure the hydrodynamic diameter and the surface charge of the biosynthesized selenium nanoparticles (SeNPs). Disposable clear zeta cells were used to measure the ultrapure water in 25 °C under the standardized conditions to determine baseline colloidal characteristics. Before analysis the suspensions of nanoparticles were sonicated gently to ensure dispersion and minimize possible aggregation. The measurements were done thrice and the mean values were reported so that there is an aspect of reliability in the analysis (Clogston and Patri, 2011).

#### 2.5.2 Transmission electron microscopy (TEM) analysis

The morphological characteristics and nanoscale architecture of the synthesized SeNPs were examined using high-resolution transmission electron microscopy (TEM; JEOL Ltd., Mitaka, Tokyo, Japan) at the Central Laboratory, Al-Azhar University. A small aliquot of the aqueous nanoparticle dispersion was briefly ultrasonicated to enhance particle distribution, and a drop was placed onto a carbon-coated copper grid for imaging. Excess liquid was removed and the sample was air-dried prior to visualization.

TEM imaging was conducted at optimized accelerating voltages to obtain representative micrographs illustrating particle morphology and approximate core size. This analysis provided direct structural confirmation of nanoscale formation and particle geometry. However, TEM observations represent localized sampling of the preparation and do not fully capture bulk dispersion characteristics; therefore, the results were interpreted in conjunction with DLS measurements to provide complementary size information.

#### 2.5.3 Fourier transform infrared (FTIR) spectroscopic analysis

FTIR spectroscopy was carried out at the Nano-Center, Faculty of Engineering, Helwan University (Cairo, Egypt) to identify the functional groups involved in the reduction, stabilization, and surface capping of the biosynthesized rutin-conjugated selenium nanoparticles (RUT-SeNPs). Spectral data were obtained using a PerkinElmer Spectrum Two FTIR spectrophotometer fitted with a Universal Attenuated Total Reflectance (UATR) diamond crystal. The spectra were recorded over the range of 4000–450 cm^-1^ at a resolution of 4 cm^-1^, averaging 10 scans per sample under ambient conditions. Air served as the background reference, and samples were placed directly on the diamond crystal to ensure uniform optical contact. The system was operated using Spectrum Two software (NIOS2 Main, Version 00.02.0091), with all analyses performed following the instrument’s calibration and internal quality control parameters. The resulting spectra were subjected to baseline correction and normalization to ensure accuracy, reproducibility, and spectral integrity for subsequent interpretation.

### 2.6 Experimental design

#### 2.6.1 Experimental animals

Forty-two healthy male albino rats, aged 21–23 days and weighing 70–80 g, were utilized in the present study were obtained from the Animal House, Faculty of Science, Helwan University, Cairo, Egypt. Animals were housed in standard polycarbonate cages equipped with stainless-steel wire lids, with no more than four rats per cage, and provided with sterilized wood shavings as bedding material. They were maintained under controlled environmental conditions, including a 12:12 h light/dark photoperiod, an ambient temperature of 22 °C ± 3 °C, and regulated relative humidity. Standard laboratory chow and fresh drinking water were supplied *ad libitum*. All husbandry practices and experimental procedures strictly adhered to internationally recognized ethical guidelines for the care and use of laboratory animals and were approved by Sinai University Research Ethics Committee (SU. REC.2025. 78 A) (1/10/2025). Animal pain or suffering was minimized as much as possible during experimentation. All processes of animal’s experimentation were executed as stated in the ARRIVE guidelines.

#### 2.6.2 Induction of SI and study design

Social isolation during childhood contributed to the SI model. Following weaning, rats were divided into two groups: social isolation-reared rats (SIR, one rat per cage) and social rearing rats (SR, 3-4 rats per cage), and the experiment lasted 60 days (Gamal El-Deen et al., 2025).

### 2.7 Experimental grouping

The experiment involved 42 animals, divided into six groups, with seven animals per group The animals were grouped as follows:

Control group (CONT, n = 7): Animals in this group were subjected to conventional settings for 60 consecutive days. At day 45, animals of this group 0.9% NaCl for 15 consecutive days.

SI group (SI, n = 7): Animals of this group were subjected to separation of each rat in separate box for 60 consecutive days. At day 45, animals of this group received 0.9% NaCl for 15 consecutive days.

SI treated with standard drug “Olanzapine” group (SI&OLA, n = 7): Animals of this group were subjected to separation of each rat in separate box for 60 consecutive days. At day 45, animals of this group received standard drug (Olanzapine) 5 mg/kg/day orally for 15 consecutive days (Kasper, 1998).

SI treated with standard drug “Rutin” group (SI&RUT, n = 7): Animals of this group were subjected to separation of each rat in separate box for 60 consecutive days. At day 45, animals of this group received Rutin 100 mg/kg/day orally for 15 consecutive days (El Wafa et al., 2020).

SI treated with the sodium selenite group (SI&Na_2_SeO_3_, n = 7): Animals of this group were subjected to separation of each rat in separate box for 60 consecutive days. At day 45, animals of this group received sodium selenite 0.5 mg/kg/day orally for 15 consecutive days (Gamal El-Deen et al., 2025; Alam-ElDein et al., 2026).

SI treated with Se nanoparticles biosynthesized using the Rutin group (SI&RUT-SeNPs, n = 7): Animals of this group were subjected to separation of each rat in separated box for 60 consecutive days. At day 45, animals of this group received RUT-SeNPs 0.5 mg/kg/day orally for 15 consecutive days (Gamal El-Deen et al., 2025).

### 2.8 Behavioral assessments

In order to determine the behavioral changes which occur in the presence of the social isolation induced schizophrenia-like phenotype, especially in negative-like symptom domains (social withdrawal, anhedonia and motivation deficits), validated paradigms that measure locomotor activity, exploratory behavior, reward sensitivity, and social affiliation were used. Light phase All behavioral tests were done under the controlled environmental conditions and apparatuses cleaned between tests to ensure elimination of olfactory cues.

#### 2.8.1 Open field test (OFT)

The open field test (OFT) was conducted to evaluate locomotor activity and exploratory behavior, which are parameters that are usually disturbed in social isolation-induced social learning models of schizophrenia and symptomatic of motivational and behavioral activation impairments. Rats were placed separately in the center of a square Plexiglas arena and were left to move about freely within the arena in a duration of 5 min. The sum of crossings (horizontal movement), the frequency of rearing (vertical search), and time of the central area were measured. The decreased locomotion and exploration drive was considered to be the behavioral aspects of dysfunction linked to negative-like symptomatology (Volosin et al., 1988; Mahmoud et al., 2025).

#### 2.8.2 Sucrose preference test (SPT)

The SP to test anhedonia was used, which is a negative-like domain of behavior commonly seen in schizophrenia-like phenotypes. Two bottles of water were habituated on the animals after which a 1% solution of sucrose was introduced in one of the bottles and the animals adapted to it after 24 h. The testing period involved 2 h of free access to a bottle of 1% solution of sucrose and a bottle of water by the rats. The preference of sucrose was based on the percentage of intake of sucrose compared to the overall fluid intake. A decrease in the preference of sucrose was explained as a decrease in the sensitivity of rewards (Liu et al., 2018; Scheggi et al., 2018).

#### 2.8.3 Social interaction test (SocInt)

The Social Interaction test (SocInt) was to measure social withdrawal and affiliative behavior specifically to evaluate the negative-like symptoms which are the hallmark of schizophrenia-like models. Two previously unknown rats belonging to the same group of the experiment were put in a neutral observation cage at the same time and given the chance to interact freely in 10 min under standardized lighting conditions. The parameters were noted as follows: Direct social contact numbers. Time of social interaction in totality (Gamal El-Deen et al., 2025).

The parameters were noted as follows: Direct social contact numbers, time of social interaction in totality, and response time to first contact. The decreased frequency and time of interaction, and the prolonged latency to respond to interaction was viewed as the loss of social affiliation due to the presence of behavioral dysfunction brought about by social isolation.

### 2.9 Tissue collection and sample preparation

In complete adherence to institutional ethical norms for animal research, all animals were humanely put to death under deep anesthesia by receiving an intraperitoneal injection of pentobarbital at a dose of 300 mg/kg. Brains were rapidly excised, and the prefrontal cortex was carefully isolated on an ice-cold glass plate. Each tissue sample was gently rinsed with chilled physiological saline to remove residual blood and ensure sample purity for subsequent biochemical and histological analyses.

A defined portion of the prefrontal cortex was homogenized in 10 mM phosphate buffer (pH 7.4) using a pre-chilled glass–Teflon homogenizer to maintain enzymatic stability and minimize oxidative degradation. The resulting homogenates were reserved for the quantitative assessment of oxidative stress indices, antioxidant defense enzymes, inflammatory cytokines, apoptotic markers, and neurochemical parameters. For the determination of monoaminergic neurotransmitters, an additional cortical aliquot was homogenized in 75% HPLC-grade methanol (10% w/v) to prevent metabolite oxidation and preserve neurotransmitter integrity. The homogenates were centrifuged at 1,358 *g* for 12 min at 4 °C, and the obtained supernatants were collected and stored at −20 °C until analysis using high-performance liquid chromatography (HPLC).

For histopathological and immunohistochemical examinations, representative samples of the prefrontal cortex were immediately fixed in 10% neutral-buffered formalin to prevent autolytic degradation. The fixed tissues were subsequently processed. Paraffin blocks were sectioned at appropriate micrometric thicknesses by employing a rotary microtome, and the sections were mounted on glass slides for routine staining and immunohistochemical evaluation under light microscopy.

### 2.10 Biochemical assays

All biochemical determinations were done using homogenates obtained from the prefrontal cortex and commercially accessible ELISA kits, precisely following the manufacturers' standardized instructions.

#### 2.10.1 Assessment of oxidative and antioxidant status

OS indices were evaluated through the quantification of biomarkers, including 8-hydroxy-2′-deoxyguanosine (8-OHdG) (Cat. No. ER1487, Wuhan Fine Biotech Co., Ltd., Wuhan, China) (Sun D. et al., 2017), MDA (Cat. No. ab118970, Abcam, Cambridge, UK) (Misra and Fridovich, 1972), and NO (Cat. No. ab65328, Abcam, Cambridge, UK) (Green et al., 1982), with absorbance readings recorded at 540 nm. The antioxidant defense profile was assessed by determining the enzymatic activities of SOD and catalase (Cat. No. ab65354 and ab8346, Abcam, Cambridge, UK) (Misra and Fridovich, 1972; Aebi, 1984), while the concentration of reduced GSH (Cat. No. ab112132, Abcam, Cambridge, UK) a key indicator of cellular redox equilibrium was measured following Ellman’s method (Ellman, 1959). All parameters were quantified according to previously validated standard protocols.

#### 2.10.2 Inflammatory/anti-inflammatory marker evaluation

The inflammatory profile of the prefrontal cortex was evaluated by quantifying the concentrations of pivotal pro-inflammatory cytokines, including TNF-α (Cat. No. NBP1-9268, Novus Biologicals, Centennial, CO, USA) and IL-1β (Cat. No. ab255730, Abcam, Cambridge, UK), using ELISA kits in accordance with the manufacturers’ standardized protocols (Abdel-Wahhab et al., 2023; Ashry et al., 2025b; 2025a).

#### 2.10.3 Apoptotic/anti-apoptotic marker analysis

Apoptotic activity within the prefrontal cortex was evaluated by quantifying the expression values of key regulatory proteins, including the anti-apoptotic marker BCL-2 (Cat. No. ER0762, Wuhan Fine Biotech Co., Ltd., Wuhan, China) and the pro-apoptotic mediators Bax (Cat. No. NBP2-69938, Novus Biologicals, Centennial, CO, USA) and Caspase-3 (Cat. No. ab39401, Abcam, Cambridge, UK). Assessments were performed employing specific ELISA kits in accordance with the manufacturers’ recommended procedures (Gad et al., 2025; Ibrahim et al., 2025).

#### 2.10.4 Monoaminergic neurotransmitter quantification

The concentrations of cortical serotonin (5-HT), dopamine (DA), gamma-aminobutyric acid (GABA), and glycine were quantified from methanolic tissue extracts using HPLC equipped with an electrochemical detection system. Infinity II liquid chromatograph Brain monoamines and related metabolites were quantified by high-performance liquid chromatography with electrochemical detection (HPLC–ECD). Tissue samples were homogenized in 0.1 M perchloric acid containing 0.01% EDTA, centrifuged (12,000 × g, 15 min, 4 °C), and the supernatants filtered (0.22 μm) prior to analysis. Separation was achieved on a reversed-phase C18 column (150 × 4.6 mm, 5 μm; [manufacturer, city, country]) maintained at 30 °C. The mobile phase consisted of 50 mM sodium phosphate buffer (pH 3.0), 0.1 mM EDTA, 1 mM sodium octyl sulfate, and 10% methanol, delivered at 1.0 mL/min. Detection was performed using a glassy carbon electrode set at +0.75 V versus Ag/AgCl reference. Quantification was based on external calibration curves generated from authentic standards (*r* ^2^ ≥ 0.995). Limits of detection and quantification were determined using signal-to-noise ratios of 3:1 and 10:1, respectively. Results were expressed as ng/g wet tissue. This analytical approach enabled precise and reliable determination of neurotransmitter levels, providing an accurate representation of monoaminergic and inhibitory neurotransmission alterations associated with SI pathology.

### 2.11 Gene expression analysis

Total RNA was isolated from prefrontal cortex samples using TRIzol® reagent (Cat No. 15596026, Thermo Fisher Scientific, USA), and complementary DNA (cDNA) was synthesized through reverse transcription of purified RNA templates using High-Capacity cDNA Reverse Transcription Kit (Cat. No. 4368814, Applied Biosystems, USA). The relative mRNA expression levels of Nrf2, MAO, and NF-κB were quantified employing gene-specific primers (Table 2).

**TABLE 2:** Primer sequences of genes analyzed by RT-qPCR.

Genes	Forward sequence	Reverse sequence	Accession number
*Nrf2*	CCT​CAG​CAT​GAT​GGA​CTT​GGA	GCG​ACT​GAA​ATG​TAG​GTG​AAG​A	NM_001399173.1
*AchE*	CCA​ATG​ACC​CTC​GAG​ACT​CTA​A	GGT​CGA​ACT​GGT​TCT​TCC​AG	NW_001084677
*MAO*	TGC​ATG​GTG​TAT​TAC​AAG​GA	CTT​GAG​ATC​CCA​GAA​CTT​TG	NM_001270458.1
*NFκB*	GTC​TCA​AAC​CAA​ACA​GCC​TCA​C	CAG​TGT​CTT​CCT​CGA​CAT​GGA​T	NM_199267.2
*GAPDH*	ATG​GTG​AAG​GTC​GGT​GTG​AAC​G	TGG​TGA​AGA​CGC​CAG​TAG​ACT​C	NM_001411843.1

GAPDH gene was employed as an endogenous control for data normalization. Relative transcript abundance was calculated using the 2^−ΔΔCT^ method, following verification of primer specificity and amplification efficiency (90%–110%) through melt curve profiling and standard curve analysis. For each target gene, biological replicates were collected from all experimental groups, and reactions were conducted in technical triplicates to ensure the robustness, precision, and reproducibility of the obtained data (Alazzouni et al., 2021; Gadelmawla et al., 2022).

### 2.12 Hormonal analysis

To assess HPA axis function, serum levels of corticosterone were measured using a sensitive ELISA kit, providing insight into stress hormone dysregulation often observed in depressive states (Lei et al., 2025).

#### 2.12.1 Assessment of glial reactivity and neurotrophic factor expression

To evaluate astrocytic activation following treatment, the expression of GFAP a well-established marker of glial reactivity was quantified in prefrontal cortex homogenates using a commercial enzyme-linked immunosorbent assay (ELISA) kit (Cat. No. NS830; Merck, Darmstadt, Germany), strictly adhering to the manufacturer’s validated protocol.

In parallel, the levels of BDNF, a critical neurotrophin involved in neuronal survival, synaptic plasticity, and cognitive modulation, were determined using an ELISA kit obtained from My BioSource (Cat. No. MBS355435; San Diego, CA, USA). All assays were performed in accordance with the supplier’s standardized technical instructions to ensure high analytical specificity, sensitivity, and reproducibility of the measurements.

### 2.13 Immunohistochemical (IHC) analysis

To evaluate astrocytic activation and neuronal survival, paraffin embedded cortical sections were subjected to IHC staining targeting glial fibrillary acidic protein (GFAP) (Cat. No. NB300-141, 1:600, Novus Biologicals, Centennial, CO, USA) and nuclear factor Kappa-B (NF-kB) (Cat. No. NB100-56712, 1:200, Novus Biologicals, Centennial, CO, USA). Serial tissue sections, cut at a thickness of 4–5 μm, were carefully deparaffinized in xylene and rehydrated. After antigen retrieval, the sections were incubated with primary antibodies against GFAP and NF-kB, then exposed to corresponding biotinylated secondary antibodies and a streptavidin peroxidase complex to amplify the signal. Visualization of immunoreactive sites was performed using 3,3′-diaminobenzidine (DAB) as the chromogenic substrate results in a noticeable brown precipitate at antigen localization sites. Following that, sections were counter-stained with hematoxylin and dried, and mounted for microscopic evaluation under bright-field illumination (Abdel-Wahhab et al., 2023; 2024; El-Gneady et al., 2025).

### 2.14 Quantitative assessment of IHC staining

This procedure serves as a reliable approach for assessing protein localization within tissue architecture. Immunohistochemical staining was analyzed in five non-overlapping fields per section selected via systematic random sampling at fixed magnification (x400). Images were captured under consistent acquisition settings. Quantitative analysis was performed using ImageJ Fiji software (version 1.2) with color deconvolution to isolate DAB staining. A uniform threshold was applied across all images for each marker, and the percentage of GFAP and NF-kB positively stained area (% area) was calculated. All image analysis was performed by an investigator blinded to group assignment to minimize bias (Alrashdi et al., 2025b; Alrashdi et al., 2025a).

### 2.15 Histopathological examination

Fixed sections of the prefrontal cortex were stained with hematoxylin and eosin (H&E) for general histopathological examination. The stained tissues were evaluated under a light microscope to assess neuronal architecture, cytoplasmic integrity, and nuclear morphology, enabling the identification of any degenerative alterations or restorative changes indicative of disease progression or therapeutic efficacy (Ibrahim et al., 2025; Lutfy et al., 2025). Hematoxylin and eosin (H&E)-stained brain sections were assessed for neuronal degeneration using a standardized semi-quantitative scoring system. Histological changes were graded on a 0–4 scale: 0 = normal architecture; 1 = minimal degeneration; 2 = mild degeneration with limited cellular alterations; 3 = moderate degeneration with evident neuronal loss; 4 = severe degeneration with extensive neuronal loss and marked structural disruption (Gibson-Corley et al., 2013). Multiple sections per animal were scored independently by a blinded investigator, and mean scores per group were used for statistical analysis.

### 2.16 Statistical analysis

All experimental data were expressed as mean ± standard error of the mean. Statistical comparisons among different experimental groups were performed using one-way ANOVA followed by Tukey’s *post hoc* test to determine intergroup significance. Prior to conducting one-way ANOVA, data normality was assessed using the Shapiro–Wilk test and homogeneity of variances was evaluated using Levene’s test. The independence of observations was ensured by the experimental design. Statistical analyses and graphical representations were carried out using GraphPad Prism software (version 9.0; GraphPad Software, San Diego, CA, USA). Differences were considered significant at a *P*-value <0.05.

## 3 Results

### 3.1 *In silico* ADME

**FIGURE 1:**
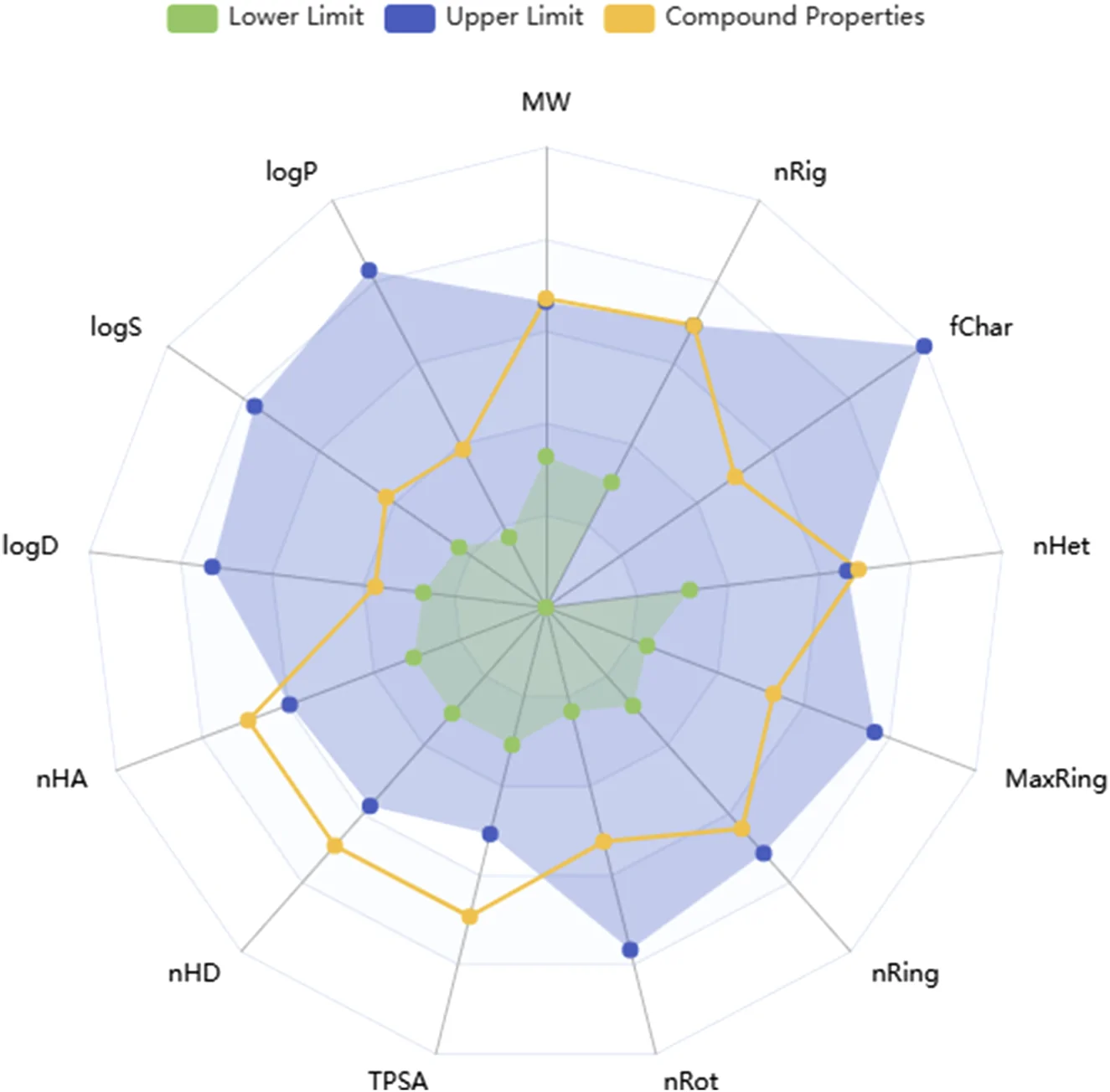
Radar of physicochemical parameters of rutin.

When evaluated using ADMETLab 3.0, rutin demonstrated promising physicochemical properties (Table 3). With the exception of Topological Polar Surface Area (TPSA) and the quantity of H-bond donors (nHD) and acceptors (nHA), rutin’s fundamental physicochemical features fall within permissible bounds. Rutin holds ten HD, sixteen HA, six rotatable bonds, and thirty rigid bonds. Rutin exhibits a significant water solubility, with a logS value of −2.4. Rutin has good membrane permeability, as indicated by its logP value of 0.98. The logD value of 1.45 demonstrates that good permeability remains preserved at physiological pH (=7.4). Figure 1 depicts the radar of the expected physicochemical features of rutin. The physicochemical properties of rutin (yellow line) do not exceed the lower limit (green line), but surpass the higher limit (blue line) in terms of nHD, nHA, and TPSA.

**TABLE 3:** Rutin physicochemical parameters.

*Physicochemical property*	*Values*
MW	610.15
nHA	16
nHD	10
TPSA	269.43
nRot	6
nRing	5
MaxRing	10
nHet	16
fChar	0
nRig	30
logS	−2.4
logD	1.45
logP	0.98

Rutin was predicted as a non-inhibitor with a high probability and a non-substrate with a medium probability for P-gp. Rutin has a high possibility of achieving bioavailability less than 50%. Rutin is projected to have an excellent volume of distribution (VDss) of 0.87 L/kg. The calculated rutin unbound fraction (Fu) in plasma was 14.6%, suggesting a medium unbound fraction. Rutin Plasma Protein Binding (PPB) showed an acceptable value of 85.0%. Significant hepatic uptake transporters include the organic anion transporting polypeptides 1B1 and 1B3 (OATP1B1 and OATP1B3). Drug interactions can occur when their normal functions are inhibited. Rutin has a projected possibility of inhibiting OATP1B1 and OATP1B3, both at 0.99. Drug-induced cholestasis is a common adverse effect of medications, typically produced by an unanticipated interaction with the bile salt export pump. Rutin was anticipated to have a poor probability of suppressing BSEP (Table 4).

**TABLE 4:** Absorption and distribution properties of rutin.

ADME property	Value
*Absorption*	
pgp_inh	6.2 × 10^−8^
pgp_sub	0.7
f50	0.99
*Distribution*	
VDss	0.87 L/kg
Fu	14.6%
PPB	85.0%
OATP1B1	0.99
OATP1B3	0.99
BSEP	0.0017

### 3.2 *In silico* toxicity

To visualize the potential toxicity profile of rutin, more toxicological research was conducted *in silico* (Table 5). Protox-3.0 organ toxicity prediction of rutin indicated nephrotoxicity, but there was also a high likelihood of non-hepatotoxic, non-neurotoxic, and non-cardiotoxic consequences. Rutin was anticipated to be immunotoxic with no signs of carcinogenicity, mutagenicity, or cytotoxicity. Rutin’s anticipated safety can be demonstrated by its inability to impact key nuclear signaling pathways, such as PPAR-α, HSE, MMP, and p53. Rutin’s safety profile was further supported by the prediction of not impacting macromolecular targets involved in molecular initiating processes, including as GABAR, NMDAR, PXR, NADHOX, and VGSC. Rutin was discovered to impede no metabolic processes.

**TABLE 5:** Toxicity prediction by Protox-3.0.

Classification	Target	Prediction	Probability
Organ toxicity	Hepatotoxicity	—	0.8
	Neurotoxicity	—	0.89
	Nephrotoxicity	+	0.77
	Cardiotoxicity	—	0.98
Toxicity end points	Carcinogenicity	—	0.91
	Immunotoxicity	+	0.98
	Mutagenicity	—	0.88
	Cytotoxicity	—	0.64
Tox21-nuclear receptor signalling pathways	Aromatase	—	0.99
	Peroxisome proliferator activated receptor gamma (PPAR-gamma)	—	0.98
	Heat shock factor response element (HSE)	—	0.99
	Mitochondrial membrane potential (MMP)	—	0.97
	Phosphoprotein (tumor supressor) p53	—	0.9
Molecular initiating events	GABA receptor (GABAR)	—	0.96
	Glutamate N-methyl-D-aspartate receptor (NMDAR)	—	0.92
	Pregnane X receptor (PXR)	—	0.92
	NADH-quinone oxidoreductase (NADHOX)	—	0.97
	Voltage gated sodium channel (VGSC)	—	0.95
Metabolism	Cytochrome CYP1A2	—	0.98
	Cytochrome CYP2C19	—	0.99
	Cytochrome CYP2C9	—	0.9
	Cytochrome CYP2D6	—	0.92
	Cytochrome CYP3A4	—	0.99
	Cytochrome CYP2E1	—	0.99

### 3.3 Molecular docking

These docking results are presented as computational predictions intended to support biological plausibility and do not constitute experimental evidence of direct target engagement. To emphasize rutin binding mechanism and interactions, molecular docking simulation was performed on targets vital to the treatment of SI, oxidative stress, and inflammation. Redocking the co-crystallized ligands of all docked targets (8ZMG, 3PBL, 3HR4, 6RLW, and 6ZEZ) yielded RMSD values of 0.936 Å, 0.543 Å, 0.763 Å, 0.300 Å, and 0.401 Å, respectively, confirming method validity. Rutin exhibited variable binding affinities to 8ZMG, 3PBL, 3HR4, 6RLW and 6ZEZ with values of −12.23, −15.90, −10.22, −11.67 and −9.35 kcal/mol, respectively (Table 6).

**TABLE 6:** Binding energy and interactions of rutin with 5-HT_2A_ receptor (PDB: 8ZMG), human dopamine D_3_ receptor (PDB ID: 3PBL), Human iNOS (PDB ID: 3HR4), OGG1 (PDB ID: 6RLW), and Keap1 (PDB ID: 6ZEZ).

PDB ID	S (Kcal/mol)	Type of interaction	Amino acid	Distance (Å)
8ZMG	−12.23	H-bond	Ser159	2.06
		Carbon-H bond	Asn363 Asp155 Asp155	2.02 2.62 2.33
		Pi-alkyl	Phe339 Val156 Val156	4.98 4.53 4.17
		Pi-lone pair	Trp336	2.74
		Pi-pi T-shaped	Phe340 Phe340 Trp336 Trp336	5.00 5.35 5.77 5.03
		Alkyl	Leu362 Leu228	4.17 4.07
3PBL	−15.90	H-bond	Asp110 Tyr373 Thr369 Glu90 Tyr36 Cys181	2.45 2.41 3.40 2.74 2.85 2.94
		C-H bond	His349	3.47
		Pi-donor H-bond	Thr369	3.54
		Pi-sulfur	Cys181	2.94
		Alkyl	Val350	5.40
		Pi-alkyl	Phe345 Phe346 Val107 Val86	4.85 3.98 4.89 4.69
3HR4	−10.22	H-bond	Arg633 Thr592 Lys549 Ser550 Glu661	6.91 4.01 2.84 4.71 5.27
		C-H bond	Gly627 Gly627 Ser591 Ser628 Ser628 Glu661 Glu661	2.92 3.40 3.82 3.16 3.68 5.20 5.27
		Pi-lone pair	Tyr631	2.86
		Pi-alkyl	Tyr631 Lys549	4.02 5.42
6RLW	−11.67	H-bond	Lys249 Lys249 Asp268 Cys253	1.95 2.05 2.47 2.66
		C-H bond	Ser147 Ser147 Ser147 Pro266	2.45 2.95 2.79 2.40
		Alkyl	Val269	5.30
		Pi-alkyl	His270 Ile152 Leu323 Leu323 Ala316	4.58 5.10 4.43 5.26 4.53
6ZEZ	−9.35	H-bond	Arg483 Ser508 Asn382 Asn414 Ser363	1.97 1.94 1.68 2.79 2.72
		C-H bond	Arg380 Ser555 Ser555	2.08 2.40 1.70
		Pi-cation	Arg415	3.70
		Pi-alkyl	Arg415 Ala556	4.68 3.85

**FIGURE 2:**
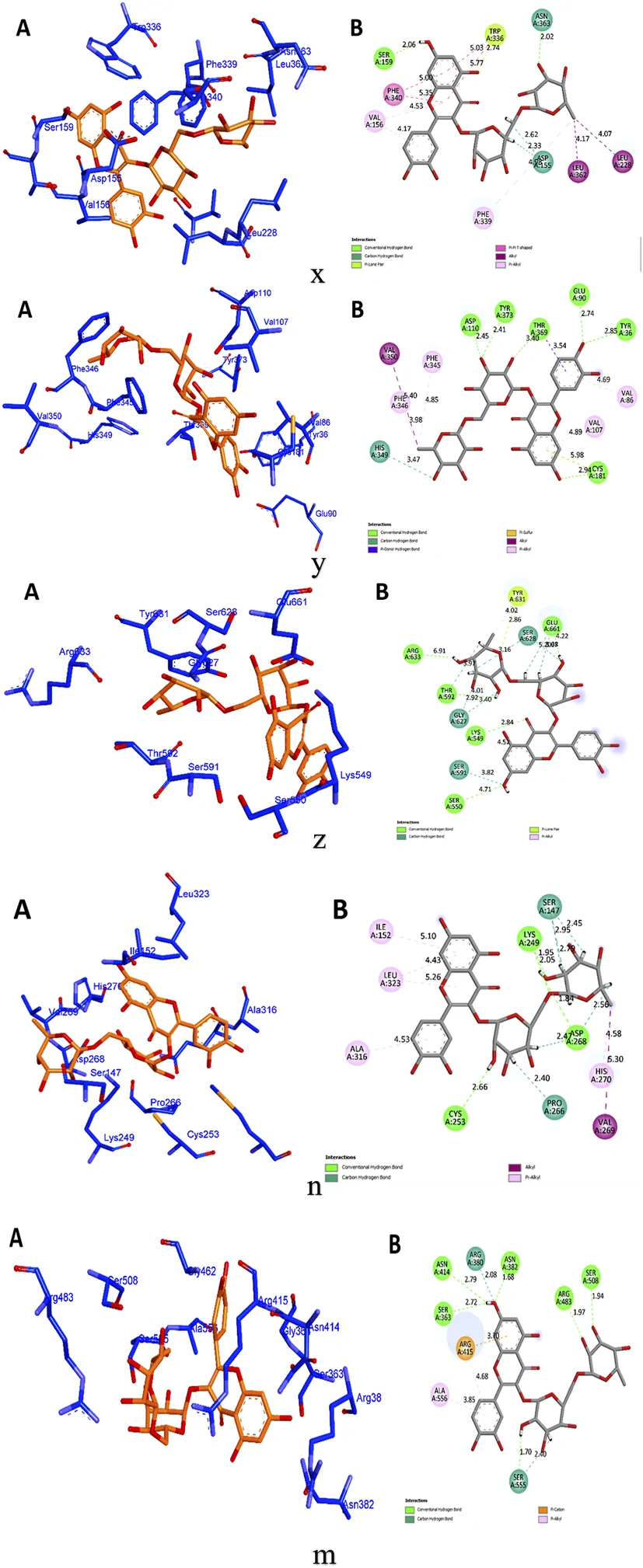
*Docking interactions of rutin with* multiple target proteins. Rutin binding modes with (X) 8ZMG, (Y) 3PBL, (Z) 3HR4, (N) 6RLW, and (M) 6ZEZ are shown. **(A)** illustrates 3D docked poses, while **(B)** presents 2D interaction diagrams highlighting key ligand–protein contacts.

Ser159 amino acid (aa) in 5-HT_2A_R was involved in a H-Bond of rutin, while Asn363 and Asp155 were involved in three Carbon-H bonds. Three pi-alkyl and two alkyl interactions were observed between rutin and 5-HT_2A_R. Four T-shaped pi-pi stacking was observed between rutin and Phe340 and Trp336 aa. One Pi-Lone pair interactions was noted between Trp336 and rutin Figure 2 (x). Such interactions could explain rutin possibility to act as inverse agonist on 5-HT_2A_ R.

In case of D_3_ receptor Figure 2 (y), rutin demonstrated six H-bonds involving Asp110, Thr373, Thr369, Glu90, Tyr36 and Cys181. One carbon-H bond was observed involving His349. One pi-H bond, pi-sulfur bond and one alkyl interaction were noted with Thr369, Cys181 and Val350, respectively. Four pi-alkyl interactions were observed between rutin and D_3_ receptor involving Phe345, Phe346, Val107 and Val86, indicating potential inhibition of D_3_ receptor.

Interaction of rutin with human iNOS resulted in five H-bonds with Arg633, thr592, Lys549, Ser550 and Glu661. Rutin demonstrated seven Carbon-H interactions with Gly627, Ser591, Ser628 and Glu661. One pi-Lone pair and two pi-alkyl interactions were observed between rutin and iNOS as illustrated in Figure 2 (z), indicating the ability of rutin to inhibit human iNOS causing a reduction in NO and further oxidative stress consequently initiated.

Lys249, Asp268 and Cys253 aa, in OGG1, were involved in four H-bonds with rutin, while Ser147 and Pro266 were involved in four Carbon-H bonds. One alkyl and five pi-alkyl interactions were observed between rutin and OGG1 illustrated in Figure 2 (n).

In case of Keap1, four H-bonds were observed between rutin and Arg483, Ser508, Asn382, Asn414 and Ser363. Three Carbon-H bonds were noted involving Arg380 and Ser555. Rutin could form one pi-cation and two pi-alkyl interactions with Keap1 Figure 2 (m), suggesting a possible inhibition of nrf2 repression.

### 3.4 Characterization of RUT-SeNPs

#### 3.4.1 Zeta potential and particle size

**FIGURE 3:**
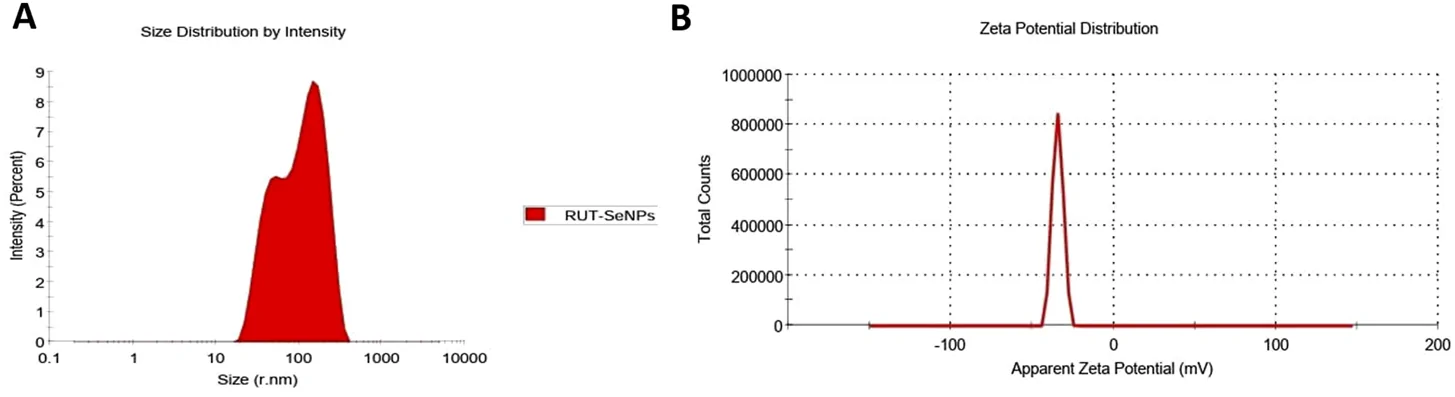
**(A)** Particles size through dynamic light scattering (DLS) and **(B)** zeta potential analyses of rutin-mediated selenium nanoparticles (RUT-SeNPs) showing nanoscale particle size distribution and a negative surface charge.

Dynamic light scattering (DLS) analysis revealed that the biosynthesized rutin-conjugated selenium nanoparticles (RUT-SeNPs) exhibited a Z-average hydrodynamic diameter of approximately 100.7 nm with a moderated interpretation of the PDI value (PDI) in range of 0.2–0.4, indicating a moderate dispersion of particle size distribution. The intensity-based size profile demonstrated two predominant peaks at 144.3 nm (71%) and 45.22 nm (29%), confirming the presence of well-dispersed nanoparticles with minimal aggregation. The zeta potential value of −34.3 mV reflected a high degree of surface charge, suggesting strong electrostatic repulsion between particles, which contributes to excellent colloidal stability of the synthesized nanostructures. The negative surface potential is primarily attributed to the abundance of hydroxyl and phenolic groups from rutin, which act as effective capping and stabilizing agents during nanoparticle formation, as shown in Figure 3.

#### 3.4.2 Transmission electron microscopy (TEM) analysis

**FIGURE 4:**
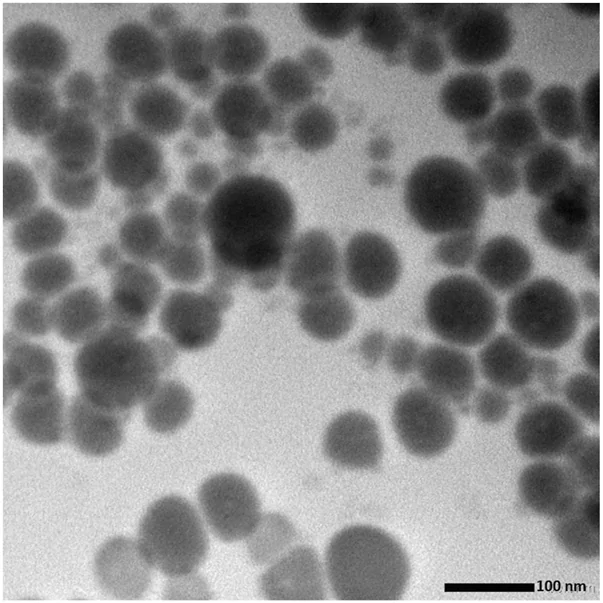
TEM micrograph of biosynthesized RUT-SeNPs showing predominantly spherical, well-dispersed particles with smooth surface morphology and uniform nanoscale distribution (scale bar = 100 nm).

TEM provided direct visualization of the morphological characteristics of the biosynthesized rutin-conjugated selenium nanoparticles (RUT-SeNPs), as shown in Figure 4. The micrograph revealed that the nanoparticles were predominantly spherical in shape with a smooth surface topology and were uniformly distributed without noticeable agglomeration. The observed nanoscale architecture confirmed the successful formation of discrete and well-dispersed particles within the size range of approximately 40–100 nm. The images demonstrated the homogeneity and structural integrity of the synthesized RUT-SeNPs, indicating effective stabilization by rutin molecules during the green synthesis process.

#### 3.4.3 Fourier Transform Infrared (FTIR) Spectroscopic analysis

**FIGURE 5:**
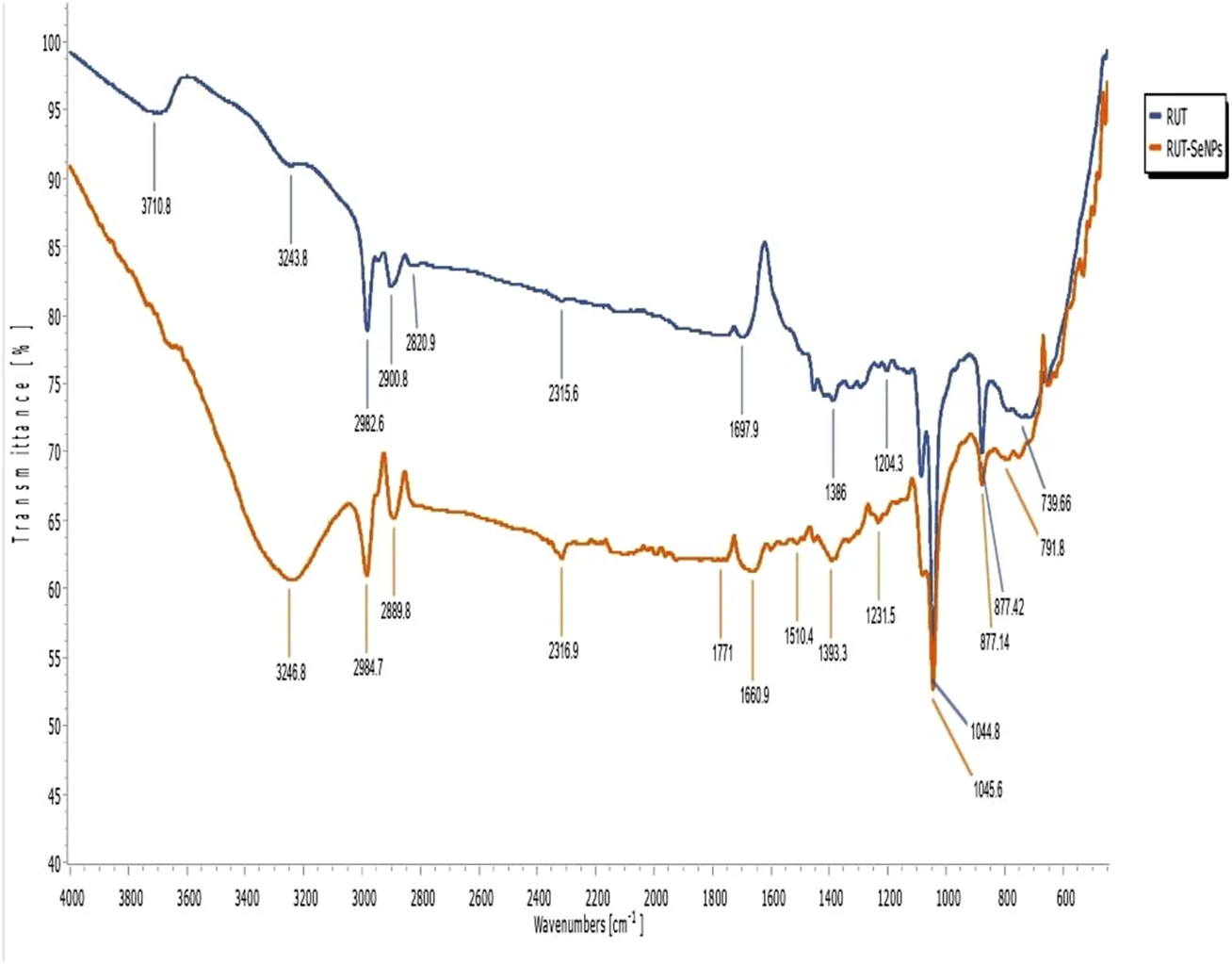
FTIR spectrum of biosynthesized rutin-conjugated selenium nanoparticles (RUT-SeNPs) showing characteristic absorption bands corresponding to hydroxyl, carbonyl, and aromatic functional groups of rutin, confirming its involvement in the reduction and stabilization of selenium nanoparticles.

The FTIR spectrum of rutin-conjugated selenium nanoparticles (RUT-SeNPs) showed a spectral change relative to free rutin, which showed the presence of functional groups in the preparation of the nanoparticles. The wide band at about 3,246 cm −1 is associated with O-H stretching vibrations of the phenolic groups indicating that they are involved in the reduction and stabilization processes. Typical peaks around 2,984–2,890 cm^-1^ are put down to C–H stretching vibration. The aromatic C=C stretching band appeared around 1,600–1,510 cm^-1^, and the peaks at 1,386–1,231 cm^-1^ are those expected of the C–O stretch of flavonoid moieties. Notably, other bands observed in the lower wavenumber region (approximately 877–739 cm^-1^ and below 600 cm^-1^) might be related to Se–O vibrations or trace elements of selenium lattice vibrations. These low frequency bands give an indication of interaction of selenium species with oxygen-containing functional groups of rutin during nanoparticles synthesis As shown in Figure 5.

### 3.5 General condition of experimental animals

During the experimental time duration, the overall health condition of the animals was critically observed. Normal control rats were found to be normal in terms of activity, grooming, and progressive increase in body weight. Conversely, physiological stress was mildly exhibited in animals in the diseased/induced group in the form of lower activity and a slight decrease in the body weight gain with the control group. Nevertheless, the overall state of the animals improved when they were treated with the compounds under investigation because, they started to show normal behavior and gradually regained the lost body weight gain. Significantly, none of the experimental groups reported any mortality or any serious adverse clinical signs throughout the study period.

### 3.6 Behavioral assessments

#### 3.6.1 Open field test and sucrose preference test

**FIGURE 6:**
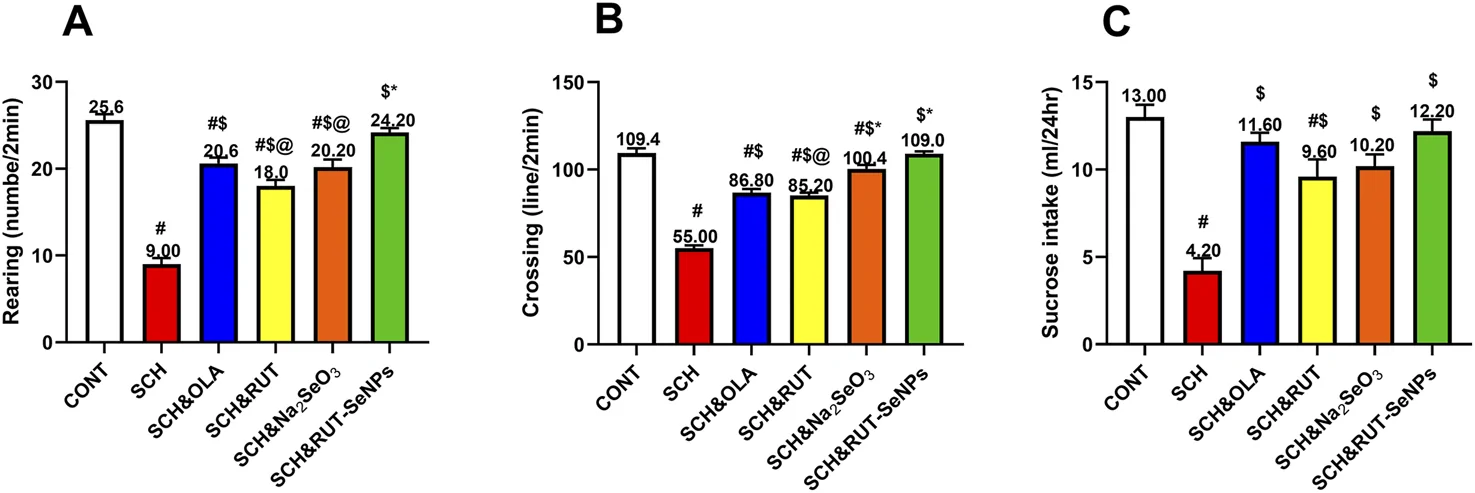
Effect of Rutin, Sodium selenite and RUT-SeNPs on in social isolation subjected rats **(A)** Rearing number, **(B)** Number of crossings in the open field test and **(C)** sucrose preference test in social isolation subjected rats t. Data is presented as mean ± SEM (*n* = 7). One-way ANOVA followed by Tukey’s *post hoc* test. # = vs. Control; $ = vs. SI (SI); @ = vs. SI&RUT-SeNPs; * = vs. SI&OLA; *P* < 0.05.

As shown in Figure 6, Induction of SI produced robust behavioral deficits across locomotors and hedonic domains. Relative to the control group, SI rats showed pronounced reductions in rearing, crossing and sucrose preference by 64.84%, 49.73% and 67.69% respectively, indicating locomotor hypoactivity, diminished exploratory drive, and anhedonia. Treatment with the standard antipsychotic olanzapine (SI&OLA) partially rescued these impairments, increasing rearing, crossings and sucrose preference by 128.89%, 57.82%, and 176.19% respectively, versus social isolation subjected group. Nevertheless, values remained below control denoting incomplete functional normalization.

The Rutin co-treated group (SI&RUT) exhibited meaningful behavioral restoration with improvements of 100% in rearing, 54.91% in crossings and 128.57% in sucrose preference relative to SI. Sodium selenite produced a greater effect on general activity and reward sensitivity, elevating the same measures by 124.44%, 82.55%, and 142.86%, respectively. Notably, co-administration of rutin-biosynthesized selenium nanoparticles yielded significant recovery with gains of 168.89%, 98.18% and 190.48% for rearing, crossing and sucrose preference respectively, versus the SI group. Also, these values were statistically comparable to normal control group and significantly superior to SI&OLA, underscoring a significantly improved impact than the commercial standard.

#### 3.6.2 Social interaction test (socInt test)

**FIGURE 7:**
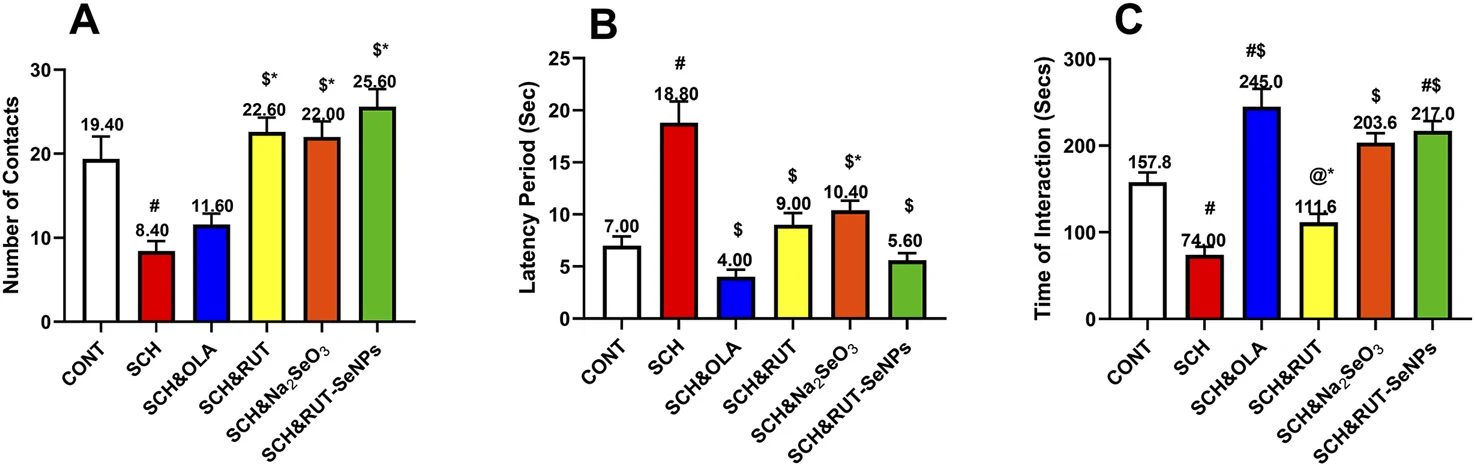
Effect of Rutin, Sodium selenite and RUT-SeNPs on **(A)** Number of Contacts, **(B)** Latency period and **(C)** Time of Interaction in the social interaction Test for SI rats. Data are presented as mean ± SEM (*n* = 7). One-way ANOVA followed by Tukey’s *post hoc* test. # = vs. Control; $ = vs. SI (SI); @ = vs. SI&RUT-SeNPs; * = vs. SI&OLA; *P* < 0.05.

As illustrated in Figure 7, SI induction elicited marked social deficits, typified by a sharp decline in the number of contacts, prolonged latency to initiate interaction, and shortened total interaction time. Compared with the control group, SI animals exhibited a 56.70% reduction in social contacts, a 168.57% increase in latency period and a 53.1% reduction in interaction duration, confirming profound social withdrawal and behavioral rigidity characteristic of SI-like pathology.

Treatment with reference antipsychotic olanzapine (SI&OLA) significantly ameliorated these deficits, increasing the number of contacts and interaction time by 38.10% and 231.08% respectively, while decreasing latency by 78.72% relative to SI animals. Nevertheless, values did not fully reach the normal control level, suggesting partial restoration. Rutin administration to SI rats produced a superior improvement pattern, increasing contact frequency and interaction time by 169.05% and 50.81% respectively, with a moderate decline in latency by 52.13% compared with social isolation subjected animals. Sodium selenite showed a comparable effect, enhancing contacts by 161.90% and interaction time by 175.14% while reducing latency by 44.68% relative to SI.

Remarkably, socially isolated animals which treated selenium nanoparticles biosynthesized using rutin (exhibited the greatest behavioral recovery. Compared with SI; the number of social contacts and interaction time improved by 204.76% and 193.24% respectively and latency period diminished by 70.21%. The SI-RUT-SeNPs group also demonstrated significantly higher contact frequency and interaction time and shorter latency than both SI&OLA and SI&RUT groups, achieving values statistically comparable to the control group.

### 3.7 Investigation of oxidative and antioxidant biomarkers

**FIGURE 8:**
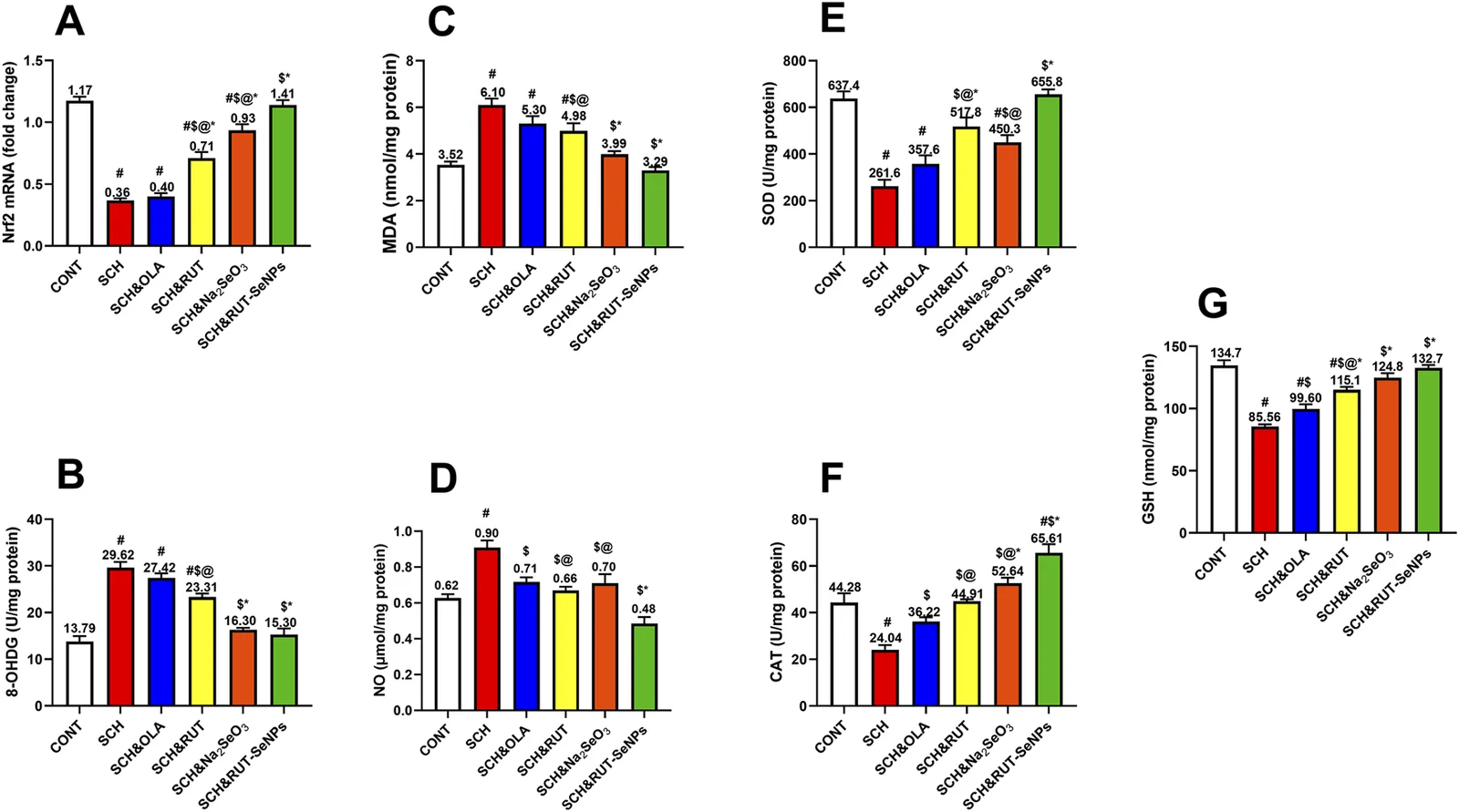
Impact of treatments on OS and antioxidant biomarkers in the prefrontal cortex. **(A)** Nrf2 mRNA fold change, **(B)** 8-OHdG, **(C)** MDA, **(D)** NO, **(E)** SOD, **(F)** CAT, and **(G)** GSH. Data are expressed as mean ± SEM (*n* = 7). One-way ANOVA followed by Tukey’s *post hoc* test. # = vs. Control; $ = vs. SI; @ = vs. SI&RUT-SeNPs; * = vs. SI&OLA; *P* < 0.05.

As depicted in Figure 8, SI induction provoked a pronounced oxidative imbalance and suppression of the endogenous antioxidant defense system. Relative to the control group, SI animals exhibited a marked downregulation of Nrf2 mRNA expression by 68.83% accompanied by significant elevations in 8-OHdG, MDA and NO levels by 114.66%, 72.90% and 44.65% respectively, reflecting enhanced oxidative DNA, lipid and nitrosative damage. In parallel, there was a substantial depletion of SOD, CAT and GSH by 58.97%, 45.70% and 36.46% compared to normal control animals, confirming oxidative distress.

Administration of olanzapine to social isolation subjected rats partially restored redox homeostasis, producing mild elevations in Nrf2 gene expression, SOD, CAT and GSH protein expression by 9.29%, 36.65%, 50.69% and 16.43% respectively; while reducing 8-OHdG, MDA and NO levels by 7.41%, 12.99% and 21.02% relative to SI group.

Co-treatment with Rutin (SI-RUT) exhibited a stronger anti-oxidative response, upregulated Nrf2 expression by 94.26% and activated the antioxidant enzymes by 97.91%, 86.83%, and 34.52% for SOD, CAT and GSH respectively; with corresponding decreases in oxidative stress biomarkers 8-OHdG, MDA and NO by 21.24%, 18.23% and 26.22% respectively; compared with SI rats.

Similarly, sodium selenite conferred substantial protection in SI rats, markedly restoring Nrf2 expression by 155.74% and increased the activities SOD by72.13%, CAT by 118.95% and GSH 45.91% while lowering 8-OHdG by 44.95%, MDA by 34.56%, and NO by 21.97% compared with SI group.

Remarkably, co-administration of SI-RUT-SeNPs displayed the most profound redox normalization. Relative to SI rats, Nrf2 mRNA expression increased by 211.48%, while 8-OHdG, MDA, and NO decreased by 48.34%, 46.00%, and 46.68%, respectively. The enzymatic antioxidants SOD, CAT, and GSH rose by 150.71%, 172.91%, and 55.09%, respectively. Compared with SI-OLA and SI-RUT; the SI-RUT-SeNPs group showed significantly higher antioxidant recovery and lower oxidative damage, achieving values nearly identical to the control group.

### 3.8 Evaluation of inflammatory biomarkers

**FIGURE 9:**
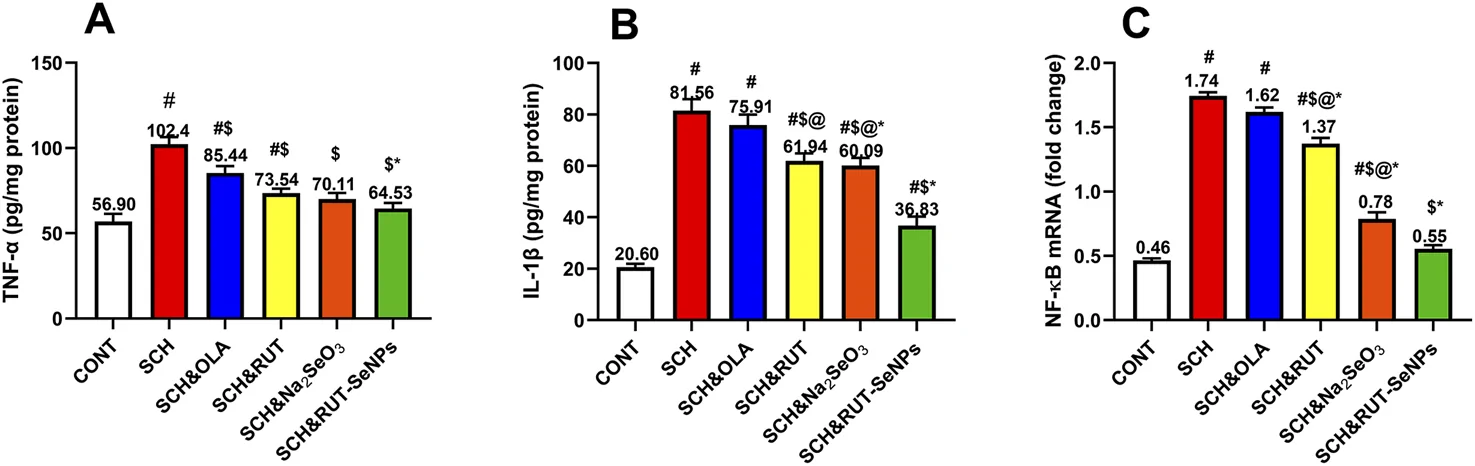
Effect of Rutin, sodium selenite, Rutin-SeNP on **(A)** TNF-α, **(B)** IL-1β and **(C)** NF-κB in the prefrontal cortex of diseased rats. Data are shown as mean ± SEM (*n* = 7). Using one-way ANOVA with Tukey’s *post hoc* test; # = vs. Control; $ = vs. SI; @ = vs. SI&RUT-SeNPs; * = vs. SI&OLA; *P* < 0.05.

**FIGURE 10:**
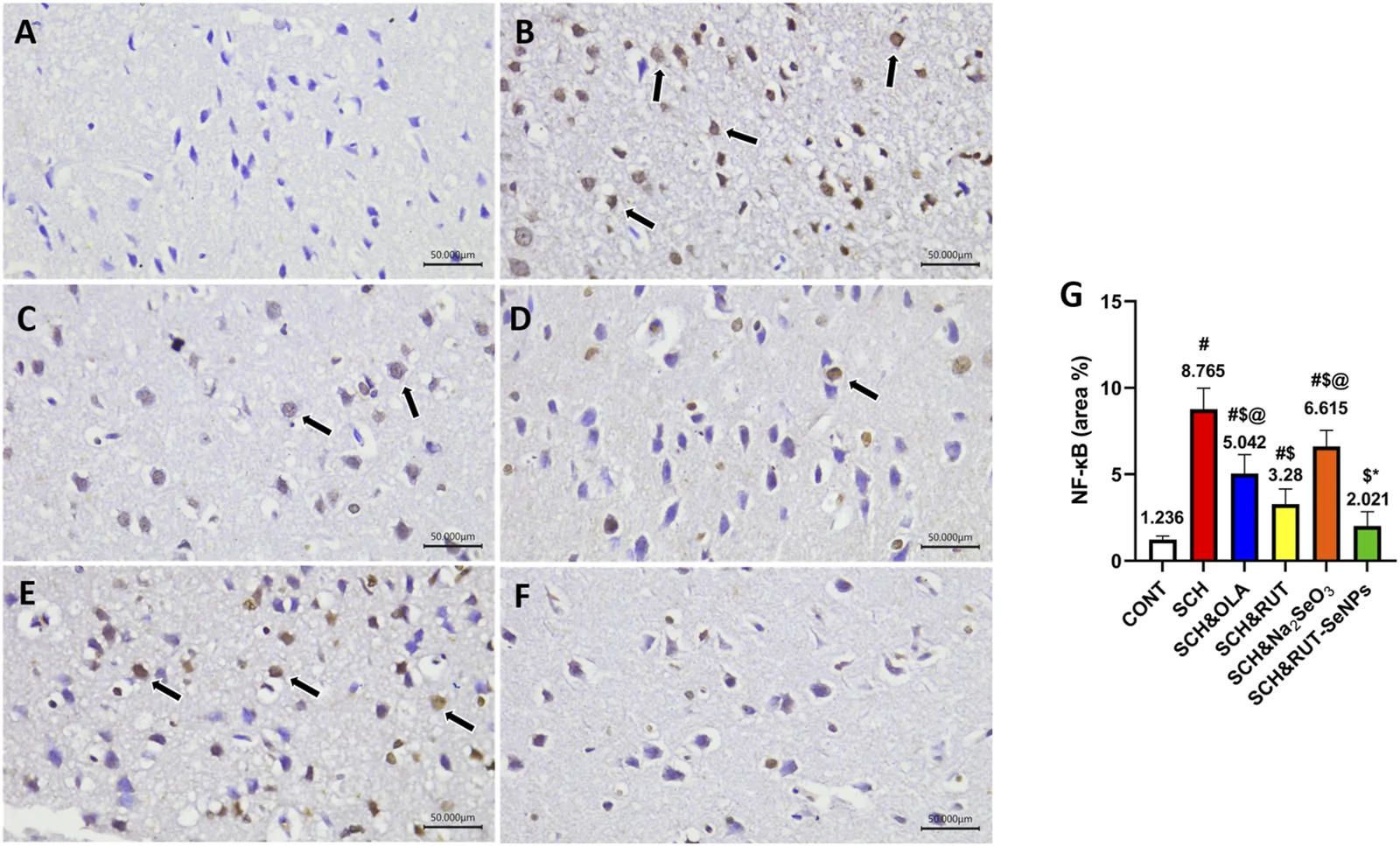
RUT-SeNPs protect against NF-κB immunoreactivity in the prefrontal cortex during SCH in rats. Photomicrographs of the prefrontal cortex from each group. NF-κB immunoreactivity was detected in tissues using DAB chromogen, resulting in a brown colour (arrow) (DAB, X400, Scale bar = 50 μm). The control group **(A)** showed normal cortical histological architecture and mild NF-κB staining. SCH **(B)** and SCH&Na2SeO3-treated **(D)** groups showed significant NF-κB immunostaining. In contrast, the SCH&OLA **(C)**, SCH&RUT **(E)**, and SCH&RUT-SeNPs **(F)** treated groups. **(G)** Quantitative analysis of immunostaining area % for NF-κB was expressed as mean ± S.E.M (n = 7). Statistical analysis by one-way ANOVA with Tukey’s post hoc test. # = vs Control; $ = vs SCH; @ = vs SCH&RUT-SeNPs; * = vs SCH&OLA; p < 0.05.

As illustrated in Figures 9, 10; SI induction elicited a profound neuroinflammatory response characterized by significant activation of pro-inflammatory mediators. Compared with the control group, SI animals showed a robust elevation in TNF-α protein expression, IL-1β protein expression and NF-κB gene expression by 79.94%, 296.07% and 273.06% respectively; confirming the induction of a potent inflammatory state associated with NF-κB-dependent cytokine cascades and neuronal distress.

Co-administration of olanzapine moderately attenuated these perturbations. Although the levels remained significantly above that of control rats; it lowered TNF-α, IL-1β, and NF-κB by 16.53%, 6.92%, and 7.05%, respectively, relative to SI group. Rutin co-treatment exhibited a stronger anti-inflammatory response in SI rats, decreasing TNF-α, IL- 1β and NF-κB by 28.18%, 24.06%, and 21.26% respectively; compared with social isolated rats, reflecting suppression of cytokine release and transcriptional activity through flavonoid-mediated inhibition of NF-κB signaling.

Likewise, sodium selenite co-administration demonstrated potent anti-inflammatory efficacy, producing marked declines in TNF-α, IL-1β −and NF-κB relative to diseased animals.

Remarkably, selenium nanoparticles biosynthesized using Rutin achieved the most pronounced modulation of inflammatory pathways when given to SI animals. Compared with the diseased group, TNF-α, IL-1β, and NF-κB expressions were reduced by 36.98%, 54.85%, and 68.17%, respectively; Furthermore, the SI-RUT-SeNPs group exhibited significantly lower cytokine and transcriptional activation levels compared with both the SI-OLA and SI-RUT groups, approaching near-control values.

IHC was used to accurately measure the transcription factor NF-κB. The control rats, social isolated rats co-treated with rutin, olanzapine, and SeNP showed weak reactivity to NF-κB, suggesting an anti-inflammatory response. However, the SI and SI-sodium selenite co-treated groups showed substantial reactivity to NF-κB, demonstrating an inflammatory pathway.

### 3.9 Evaluation of apoptotic biomarkers

**FIGURE 11:**
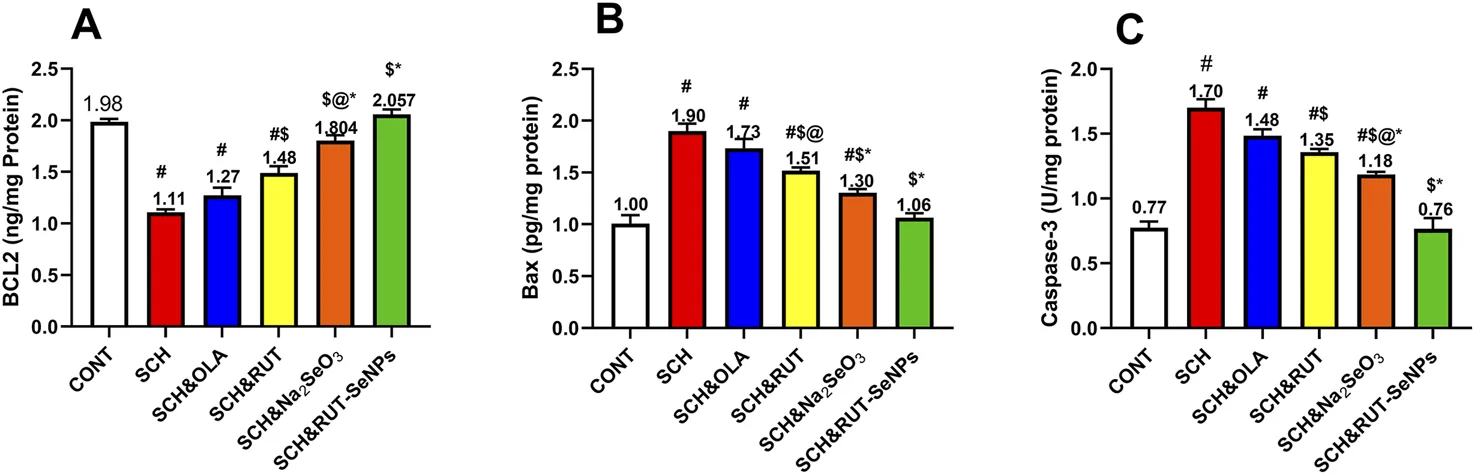
Effect of Rutin, sodium selenite, Rutin-SeNP on **(A)** BCL-2, **(B)** Bax, and **(C)** Caspase-3 expression in the prefrontal cortex of social isolated rats. Data are expressed as mean ± SEM (*n* = 7). Using One-way ANOVA followed by Tukey’s *post hoc* test; # = vs. Control; $ = vs. SI; @ = vs. SI&RUT-SeNPs; * = vs. SI&OLA; *P* < 0.05.

As illustrated in Figure 11, SI induction provoked a clear apoptotic imbalance, typified by a pronounced elevation in the pro-apoptotic markers (B) Bax by +88.9% and (C) Caspase-3 by +119.9%, accompanied by a marked suppression of the anti-apoptotic marker (A) BCL-2 by −44.1% compared with the control group (CONT, *P* < 0.05). These alterations signify activation of the intrinsic apoptotic pathway and neuronal vulnerability under chronic stress conditions.

Administration of olanzapine (SI&OLA) exerted a modest anti-apoptotic effect, decreasing Bax −8.79% and Caspase-3 −12.8%, while slightly elevating BCL-2 expression +14.5% relative to SI (*P* < 0.05). Treatment with rutin (SI&RUT) produced a more potent response, suppressing Bax and Caspase-3 by 20.2% and 20.3%, respectively, and restoring BCL-2 by 34.2% compared with SI. Likewise, sodium selenite (SI&Na_2_SeO_3_) offered substantial protection, downregulating Bax −31.4% and Caspase-3 −30.3% while enhancing BCL-2 by +62.6% relative to SI.

Remarkably, animals treated with selenium nanoparticles biosynthesized using rutin (SI&RUT-SeNPs) demonstrated significant improvement of apoptotic signaling. Compared with SI, Bax and Caspase-3 were significantly reduced by 44.0% and 55.0%, respectively, while BCL-2 increased by 85.3%. Values of Bax and Caspase-3 in the SI&RUT-SeNPs group approximated those of CONT, with BCL-2 slightly surpassing control levels +3.5%. Furthermore, SI&RUT-SeNPs exhibited superior efficacy compared to both SI&OLA and SI&RUT groups, underscoring its potent anti-apoptotic potential and neuroprotective advantage.

### 3.10 Evaluation of neurotransmitter biomarkers

**FIGURE 12:**
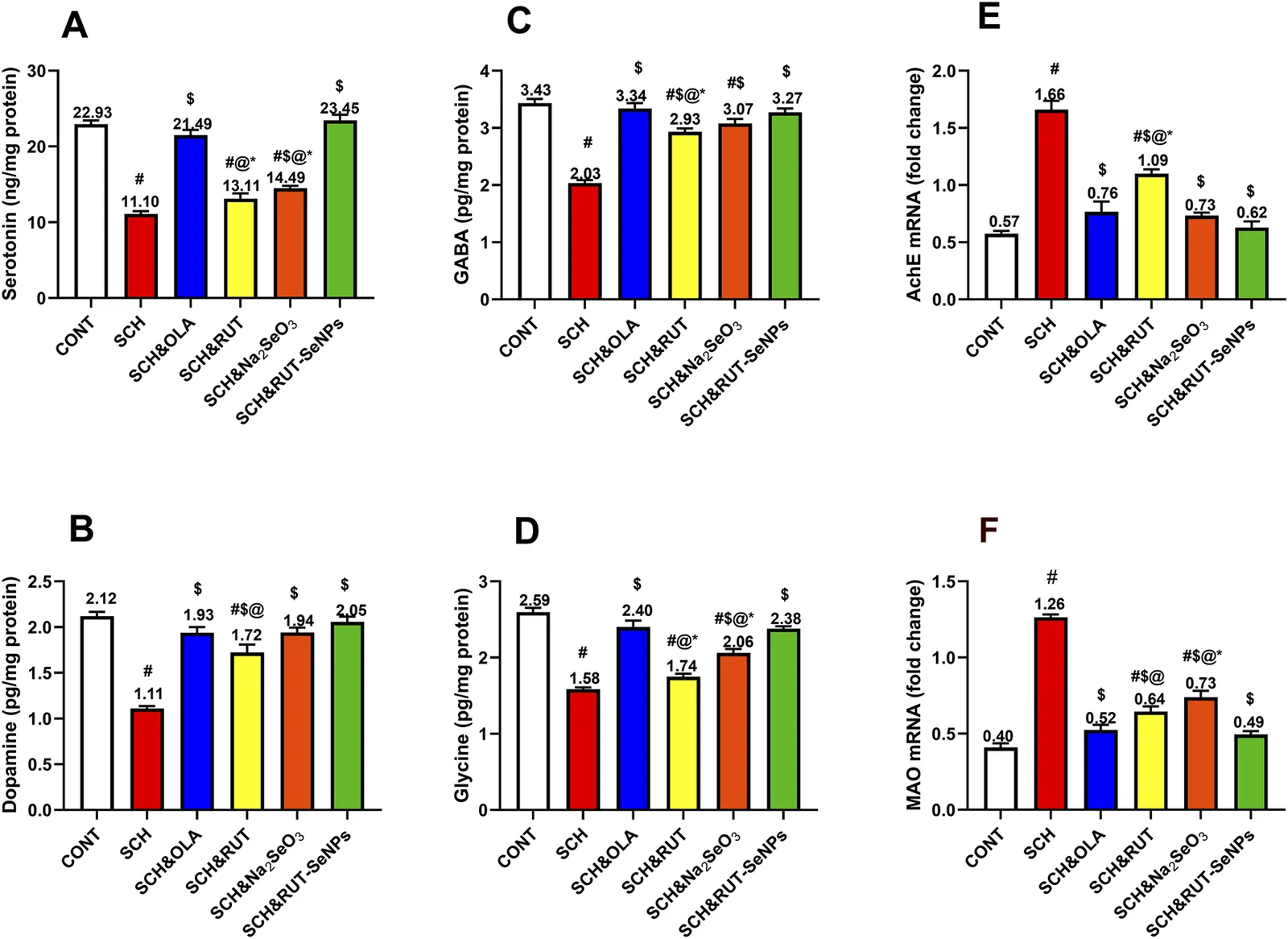
Effect of Rutin, sodium selenite, Rutin-SeNP on **(A)** Serotonin, **(B)** Dopamine, **(C)** GABA, **(D)** Glycine, **(E)** AChE and **(F)** MAO in the prefrontal cortex of SI animals. Data are expressed as mean ± SEM (*n* = 7). We used One-way ANOVA followed by Tukey’s *post hoc* test. # = vs. Control; $ = vs. SI; @ = vs. SI&RUT-SeNPs; * = vs. SI&OLA; *P* < 0.05.

As presented in Figure 12, SI induction induced profound neurochemical disequilibrium, reflecting the hallmark monoaminergic and inhibitory neurotransmission deficits. Relative to the control group, SI animals exhibited marked decreases in serotonin, dopamine, GABA and glycine by 51.57%, 47.55%, 40.78% and 38.94% respectively, accompanied by significant elevations in the catabolic enzyme acetylcholinesterase AChE by 189.37% and monoamine oxidase MAO by 208.55%. These alterations reflect neurotransmitter exhaustion and enzymatic hyperactivity underlying SI-associated cognitive and behavioral impairments.

Administration of olanzapine yielded partial neurochemical restoration, with significant increases in serotonin, dopamine, GABA and glycine by 93.64%, 74.26%, 64.27% and 51.55% respectively, while AChE and MAO activities decreased by 53.91% and 58.55%, respectively, relative to SI. Co-administration of Rutin (SI-RUT) produced moderate amelioration, elevating serotonin by 18.15%, dopamine by 54.83%, GABA by 44.06%, and glycine by 10.28% while lowering AChE and MAO activities by 33.79% and 48.89%, respectively, compared with SI.

Similarly, sodium selenite administration to social isolated rats markedly improved neurotransmission balance, enhancing serotonin, dopamine, GABA and glycine by 30.59%, 74.59%, 51.31% and 29.96% respectively, while suppressing AChE and MAO by 55.84% and 41.54%, respectively, versus diseased rats.

Strikingly, rats co-treated with selenium nanoparticles biosynthesized using rutin (SI-RUT-SeNPs) shows significantly improved of neurochemical parameters. Compared with SI rats, serotonin, dopamine, GABA, and glycine increased by 111.23%, 85.06%, 60.98%, and 50.09%, respectively, while AChE and MAO activities declined by 62.23% and 60.91% respectively, Furthermore, SI-RUT-SeNPs co-treated animals displayed significantly higher monoamine and inhibitory neurotransmitter levels and lower catabolic enzyme expression compared with SI- olanzapine and SI-Rutin groups.

### 3.11 Evaluation of neuronal and glial function biomarkers

**FIGURE 13:**
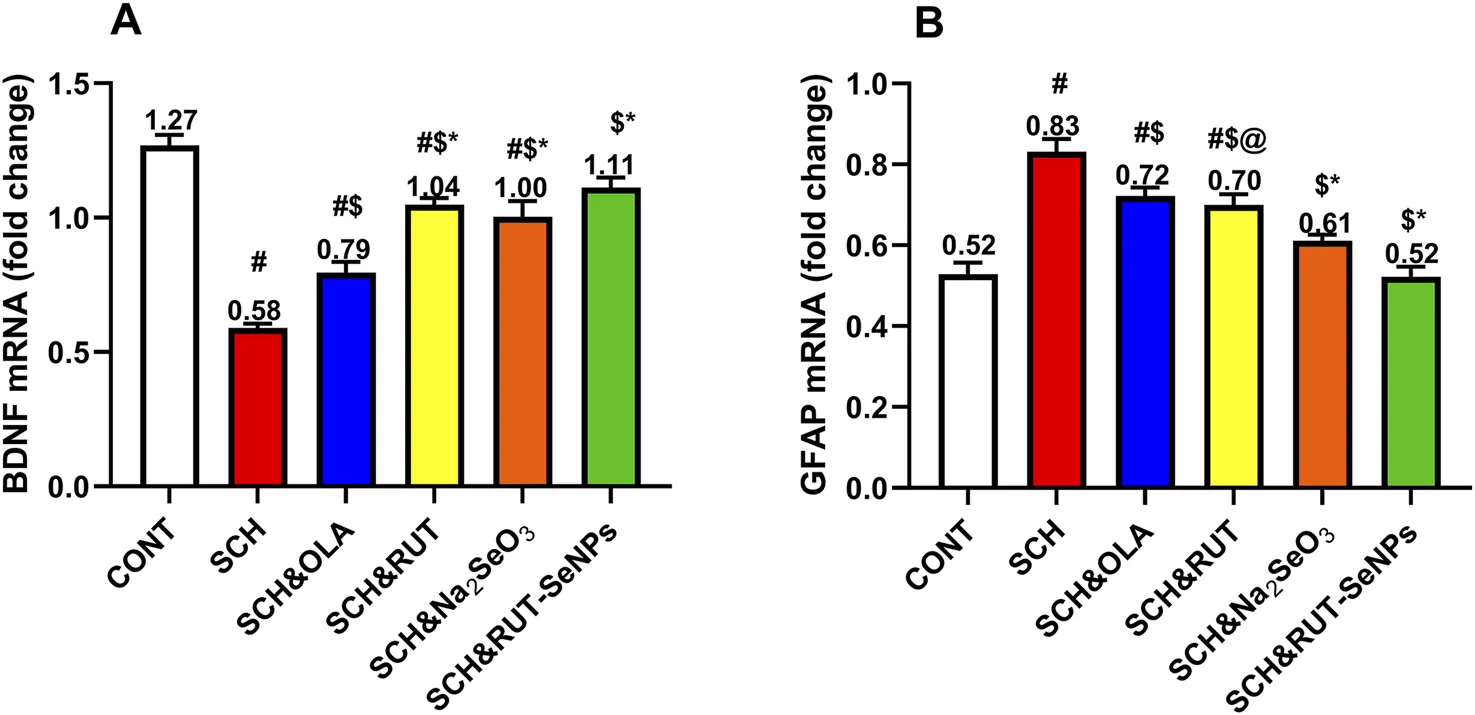
Effect of treatments on neuronal and glial function biomarkers in the prefrontal cortex. **(A)** BDNF mRNA fold change and **(B)** GFAP mRNA fold change. Data are represented as mean ± SEM (*n* = 7). One-way ANOVA followed by Tukey’s *post hoc* test. # = vs. Control; $ = vs. SI; @ = vs. SI&RUT-SeNPs; * = vs. SI&OLA; *P* < 0.05.

**FIGURE 14:**
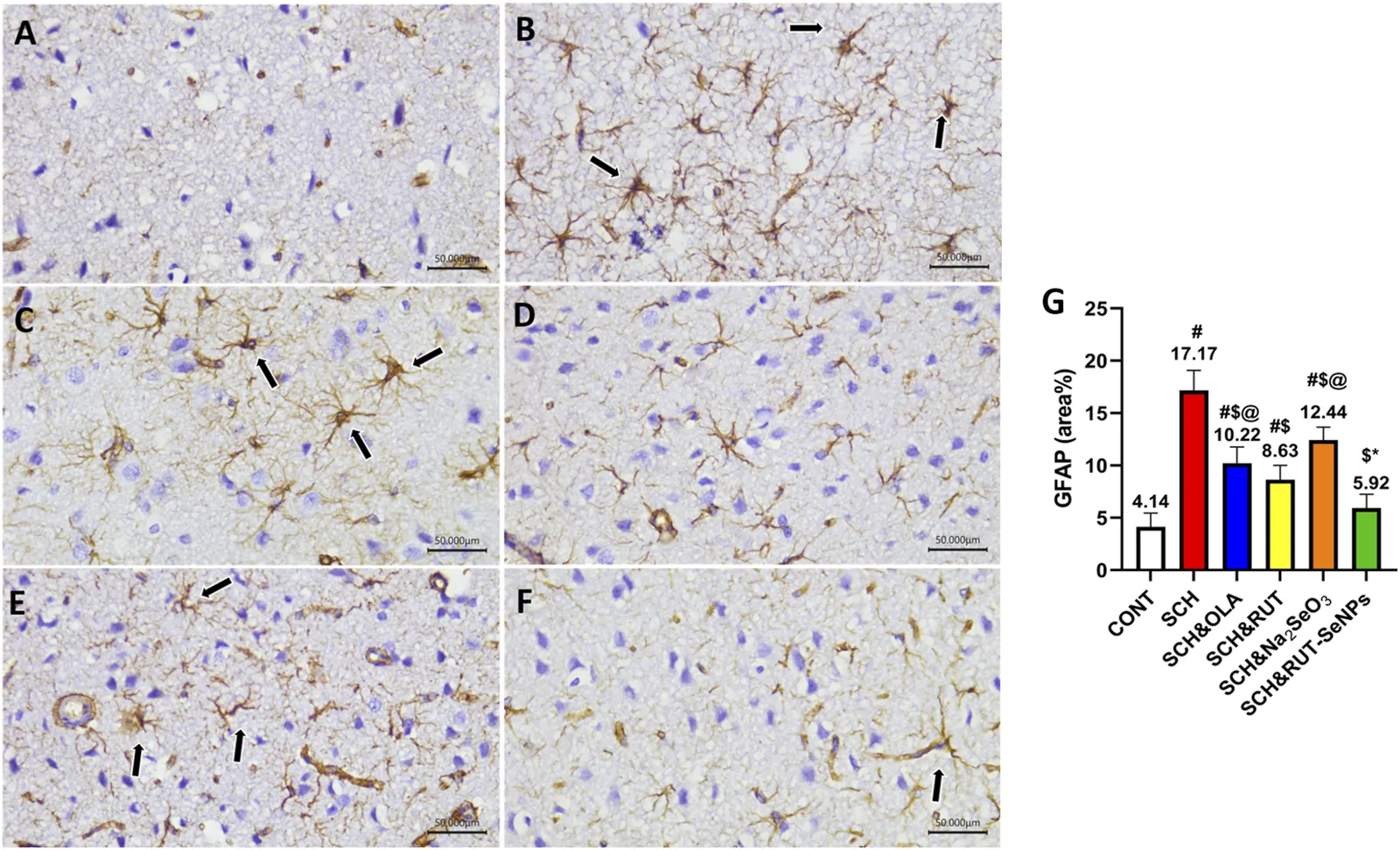
RUT-SeNPs protect against GFAP staining in the prefrontal cortex of rats with SI. Photomicrographs of the prefrontal cortex from each group. GFAP immunoreactivity was detected in tissues using DAB chromogen, resulting in a brown color (arrow) (DAB, X400, Scale bar = 50 µm). The control group **(A)** had normal cortical anatomy and mild GFAP staining. The SI **(B)** and SI&Na2SeO3-treated **(E)** groups showed strong GFAP immunostaining. In contrast, the **(C)** SI&OLA, **(D)** SI&RUT, and **(F)** SI&RUT-SeNP-treated groups demonstrated decreased GFAP staining. **(G)** Quantitative analysis of immunostaining area % for GFAP was expressed as mean ± S.E.M (*n* = 7). Statistical analysis by one-way ANOVA with Tukey’s *post hoc* test. # = vs. Control; $ = vs. SI; @ = vs. SI&RUT-SeNPs; * = vs. SI&OLA; *P* < 0.05.

As shown in Figures 13, 14, SI induction produced pronounced neurodegenerative alterations, reflected by a substantial reduction in brain-derived neurotrophic factor (BDNF) and a concurrent increase in glial fibrillary acidic protein (GFAP) relative to the control group (CONT). Specifically, BDNF declined by 53.59%, denoting severe disruption of neuronal trophic signaling and synaptic maintenance, while GFAP increased by 57.32%, indicative of astroglial activation and neuroinflammatory reactivity characteristic of SI pathology.

Treatment with olanzapine (SI-OLA) elicited partial neurorestoration, elevating BDNF by 35.13% and reducing GFAP by 13.20% compared with rats subjected to social isolation. Similarly, rutin (SI-RUT) co-administration improved neuronal integrity, enhancing BDNF expression by 77.90% and lowering GFAP by 15.82% relative to diseased group. Sodium selenite also afforded a strong protective response, increasing BDNF by 70.33% while suppressing GFAP by 26.50% compared with SI rats.

Remarkably, diseased rats co-administered selenium nanoparticles biosynthesized using rutin exhibited the most profound neuroprotective outcome. Relative to diseased, BDNF levels were elevated by 88.70%, whereas GFAP expression decreased by 37.18%. Additionally, this group demonstrated significantly higher BDNF and lower GFAP values than both the SI-OLA and SI-RUT groups, achieving values statistically comparable to the control group.

For more accuracy, the transcriptional factor, GFAP, was estimated by immunohistochemistry. The control rats, rats co-treated with rutin, rats co-treated with olanzapine and rats co-treated with SeNP expressed weak reactivity to GFAP. whereas SI group and SI-Na_2_SeO_3_-co-treated group displayed a marked reactivity to GFAP.

### 3.12 Histopathological assessment

**FIGURE 15:**
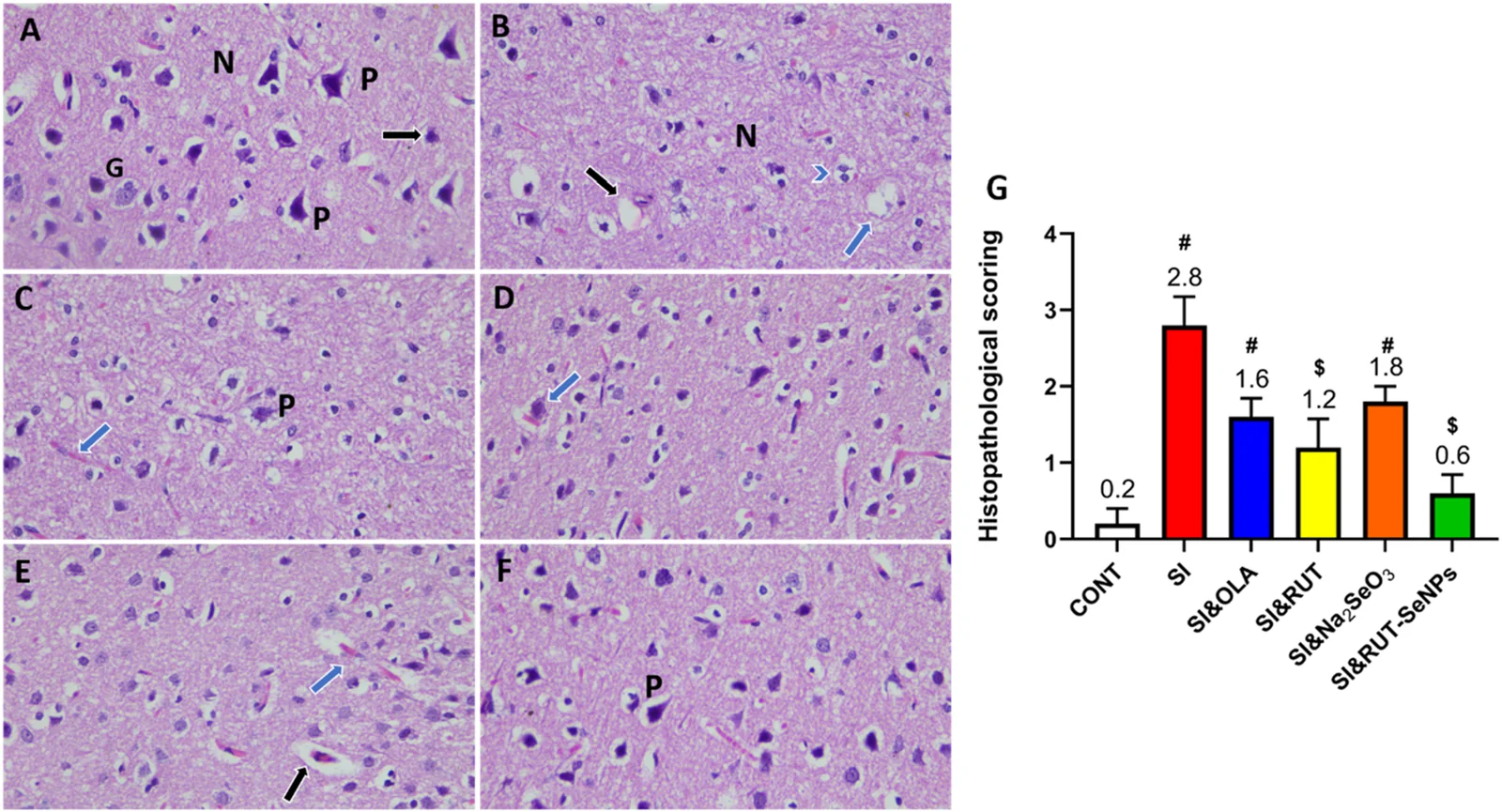
Photomicrographs of male prefrontal cerebral cortex of all groups. **(A)** Control group showed normal neuropil (N) containing vesicular pyramidal cells (P) and multiple neuroglial cells (black arrow). **(B)** SI revealed degenerated neuropil (N) with many spaces, congested blood capillaries, deformed neurons with pyknotic nucleus surrounded by halos (blue arrow head), and increased neuroglial cells (black arrow). **(C)** SI&OLA-showed degenerated pyramidal cell (P) and congested capillaries (blue arrow). **(D)** SI&RUT exhibited normal neuropil with degenerated neuron (blue arrow). **(E)** SI&Na_2_SeO_3_ showed degenerated cells with pyknotic nuclei (black arrow) and congested capillaries (blue arrow). **(F)** SI&RUT-SeNPs showed normal cortical tissue structure with normal pyramidal cells (P). (H&E, X400, Scale bar = 50 µm). **(G)** Semi-quantitative histopathological analysis of all experimental groups was expressed as mean ± S.E.M (*n* = n). Statistical analysis by one-way ANOVA with Tukey’s *post hoc* test. # = vs. Control; $ = vs. SI; @ = vs. SI&RUT-SeNPs; * = vs. SI&OLA; *P* < 0.05.

Sections of the prefrontal cortex from male control rats showed normal histological organization. Medium-sized pyramidal neurons with triangular cell bodies, basophilic cytoplasm, rounded nuclei, and distinct apical dendrites were evident. The neuropil appeared as a fibrillar eosinophilic background formed by a dense network of branching processes containing glial cells. Conversely, the SI group exhibited disrupted cortical architecture with an increased number of glial cells. Pyramidal neurons displayed degenerative changes, including neuronal distortion, darkly stained nuclei, perineuronal halos, and widening of the perinuclear space. Both SI&OLA and SI-RUT groups revealed normal architecture with minimal degenerated neurons and congested capillaries. The SI–Na_2_SeO_3_–treated group exhibited degenerative changes characterized by pyknotic nuclei, congested capillaries, and the presence of a few deformed neurons, along with occasional neurons surrounded by clear perineuronal spaces. In contrast, rats treated with SI–RUT–SeNPs showed an almost normal cortical architecture, with preserved neuronal morphology and an intact neuropil. The semi-quantitative analysis showed marked variance between SI group and SI&RUT and SI&RUT-SeNPs groups (*P* = 0.0062 and 0.0002) and the administration of RUT and RUT-SeNPs on SI rats showed remarkable improvement nearly as control (Figure 15).

## 4 Discussion

The current experiment used a social isolation rearing paradigm as an authenticated experimental model which recreates schizophrenia-like phenotypes especially negative-like behavioral domains (Powell and Swerdlow, 2023). Even though some behavioral tests utilized in this research can as well be utilized in a study relating to stress or depression, the overall behavioral, neurochemical, inflammatory, and apoptotic data points to an interpretation that is in a mechanistic system of schizophrenia and not a classical system of depression (Sun et al., 2017b; Ayilara and Owoyele, 2023). Although several parameters approached control values, the observed improvements represent statistically significant treatment effects, consistent with the inherent biological variability of complex neuropsychiatric models.

Importantly, the superior efficacy observed with rutin-conjugated selenium nanoparticles should be interpreted within the context of a comparative functional framework rather than as definitive proof of nano-specific pharmacokinetic superiority. The enhanced neuroprotective profile of RUT-SeNPs relative to free rutin and sodium selenite may plausibly reflect improved molecular stability, enhanced cellular uptake, and coordinated redox modulation resulting from nano-conjugation. However, as pharmacokinetic distribution and tissue bioavailability were not directly assessed in the present study, the observed advantages are best described as functional outcomes associated with nano-formulation, rather than mechanistically proven nano-delivery effects.

On a molecular scale, it is increasingly becoming possible that environmental stressors can be connected to schizophrenia-related pathogenesis via an early-life N-methyl-D-aspartate receptor (NMDAR) hypofunction. The previous study hypothesized that NMDAR impairment increases disruption of redox homeostasis, which causes amplification of oxidative stress and subsequent synaptic dysfunction (Momtazmanesh et al., 2019). This oxidative dysregulation may also increase neuroinflammatory cascades, so redox imbalance and immune activation may interrelate with each other. The model offers a biologically viable linkage between SI exposure and oxidative stress and inflammatory signaling and behavior dysfunction (Hardingham and Do, 2016).

OS-provoked neuronal malfunction has been regarded an important element in the genesis of SI (Yang et al., 2017). At the molecular level, these pathways are expected to incorporate hereditary reasons that enhance an individual’s susceptibility to OS and results in gene expression disruption triggered by aberrant regulation of redox-sensitive transcription (Ermakov et al., 2021). Additionally in SI; OS values have risen as evidenced by a rise in the level of harmful reactive species and a decrease in anti-oxidant defenses for fighting them. Evidence suggests that a main component of SI could be increased OS, which contributes to the worsening progression and adverse effects correlated with SI (Murray et al., 2021).

Nrf2 is a major regulator of antioxidant response and a potential treatment option for neurodegenerative conditions. Under OS, Nrf2 does not degrade normally in the cytoplasm but instead travels to the nucleus, where it attaches to a DNA promoter and promotes the transcription of anti-oxidative genes. Nrf2 upregulation is connected to elevated cellular glutathione levels and regulates numerous antioxidant enzymes to preserve balance. Given its important function in controlling the cellular antioxidant response, upregulation of Nrf2 has been proposed as a prevalent treatment option in neuropsychiatric conditions such as major depressive disorder and SI, which have been linked to chronic OS and nitrosative stress, characterised by rising concentrations of ROS and NO (Bhandari et al., 2021; Morris et al., 2021; Zhu et al., 2023; Elhemiely et al., 2025).

Elevated amounts of damaging ROS and decreased antioxidant defence mechanisms are signs of higher OS, which is linked to SI. Previous study has indicated that persons with SI, including non-medicated, medicated, first-episode, and chronic patients, had lower quantities of total antioxidants and GSH. Furthermore, they have decreased antioxidant enzyme levels such as CAT and SOD in their brain tissue (Khan et al., 2024).

The formation and accumulation of ROS may cause oxidative damage in the DNA, resulting in DNA/protein-related diseases such as neurodegeneration. Thus, the biological value of changes in 8-OHdG levels has been examined in a variety of disorders (Korkmaz et al., 2018).

Accordingly in our study we found that SI model in rats showed oxidative stress in prefrontal cortex which appeared as downregulated Nrf2 gene expression, GSH, CAT and SOD protein expression along with increased expression of MDA, NO and 8-OHdG as previously mentioned by Colaianna et al. (2013), Wang et al. (2021), Gamal El-Deen et al. (2025).

As selenium is an essential trace element with recognized antioxidant and neuroprotective roles, primarily mediated through selenoproteins involved in redox regulation; however, it also exhibits a relatively narrow therapeutic window between physiological requirement and toxicity. Excessive selenium exposure has been associated with neurological and systemic adverse effects, underscoring the importance of careful dose selection and formulation strategy. In the present study, the selenium dose was selected based on prior *in vivo* evidence demonstrating biological efficacy without overt toxicity, and no clinical signs of selenium intoxication were observed during the experimental period (Mamun et al., 2024). Notably, nano-formulation has been proposed to enhance selenium bioavailability while potentially reducing nonspecific systemic exposure, although comprehensive toxicological evaluation remains essential for translational application (Pilon-Smits et al., 2017; Razzaque and Wimalawansa, 2025), also improves the translational aspect of the plan. It was demonstrated that selenium nanoparticles demonstrated better redox activity, bioavailability, and decreased systemic toxicity in comparison with the traditional selenium salts (Raturi et al., 2025).

On top of alterations of behavior, SI rearing results in a large-scale biological change, such as mitochondrial impairment, immune deregulation, oxidative disequilibrium, and neurochemical disruptions (Möller et al., 2013). On the other hand; Rutin exhibited anti-oxidative stress effect in form of upregulated Nrf2 gene expression, GSH, CAT and SOD protein expression accompanied by elevated MDA, NO and 8-OHdG expressions (Çelik et al., 2020; Oshodi et al., 2021; Emudainohwo et al., 2023; Saafan et al., 2023). Also, sodium selenite abrogates SI behavior induced oxidative stress damage by increasing Nrf2 gene expression, GSH, CAT and SOD protein expression together with decreasing MDA, NO and 8-OHdG expressions; these data are resemble those obtained by Alrashdi et al. (2023), Shu et al. (2025), Gamal El-Deen et al. (2025) and finally selenium nanoparticles biosynthesized using Rutin achieved the most pronounced modulation of oxidative stress as it greatly increased the transcriptional factor; Nrf2 with its downstream GSH, CAT and SOD with pronounced decrease in MDA, NO and 8-OHdG expressions as described by Shalaby et al. (2023), Mohamed et al. (2023), this noticed anti-oxidant effect may be linked to the increased levels of Nrf2, which is considered a common target for treatment in neuropsychiatric conditions such as major depressive disorder and SI. Various Nrf2 modulators are now in clinical trials and may assist with minimising OS and neuroinflammation (Bhandari et al., 2021; Morris et al., 2021).

In line with this model, the changes in the inflammatory cytokines have been reported several times to be involved in the pathology of schizophrenia. Previous study identified the presence of consistent abnormalities in pro-inflammatory cytokines, which contributes to immune dysregulation as an additional contributory factor in behavioral and cognitive abnormalities. NF-kB signaling is an important regulatory hub in these inflammatory pathways, which coordinates cytokine synthesis and oxidative stress. Polyphenols and flavonoids have been reported to regulate NF-kB activity and inhibit neuroinflammatory response (Momtazmanesh et al., 2019).

Social isolation was strongly linked with elevated inflammation; thus, inflammation could play an essential part in the rapid physical ageing seen in SCH (Lee et al., 2017; Matthews et al., 2024). In the prefrontal cortex of adult male rats; social isolation causes decreased glutathione causing oxidative stress. Social isolation leads to a shift in the prooxidant-antioxidant balance, activating NF-κB. This boosts the production of genes which lead to the induction of oxidative/inflammatory mediators (Filipović et al., 2017). So, neuroinflammation, particularly in the prefrontal cortex, is well documented in a subset of SCH patients, with large elevations in inflammatory markers such as cytokines. SCH cases with cortical inflammation may have a disrupted NF-κB pathway in the brain, which can suppress normal inflammatory responses and anti-inflammatory processes (Murphy et al., 2020; 2021). Although social isolation is often associated with hyperlocomotion in certain paradigms, prolonged or developmental isolation has also been reported to induce reduced exploratory activity and motivational deficits, consistent with social withdrawal and anhedonia-related phenotypes (Gamal El-Deen et al., 2025).

Once NF-κB is stimulated, pro-inflammatory cytokines as TNF-α and IL-1β are activated explaining that increased TNF-α and IL-1β have a main impact in cognitive impairment (Hennessy et al., 2017; Taoro-González et al., 2019).

In our investigation, pro-inflammatory biomarkers in the prefrontal cortex of the social isolation subjected-rats showed a significant increase in the transcription factor NF-κB and pro-the inflammatory TNF-α and IL-1β expression as previously concluded by Gamal El-Deen et al. (2025), Alshammari et al. (2020).

To underscore this argument, the structural analogs of rutin, flavonoids have been shown to have the ability to modulate the brain cytokine signaling, as well as enhance behavioral performances in experimental models. As an example the quercetin prevented interleukin-mediated neuroimmune changes and alleviated abnormalities in the behavior of rodents (Kılıç et al., 2025).

In contrast, Rutin overcome this neuroinflammation by downregulation of TNF-α, IL-1β and NF-κB (Çelik et al., 2020; Chang et al., 2022). Additionally, sodium selenite showed anti-inflammatory effect which revealed as decreased TNF-α, IL-1β and NF-κB expressions (Li et al., 2022; Shu et al., 2025).

Great importance given to Selenium nanoparticles which reduced neuroinflammation in prefrontal cortex tissue which evidenced by the declined generation of the transcription factor NF-κB and TNF-α and IL-1β (Albrakati et al., 2021; Mohamed et al., 2023; Lutfy et al., 2025).

This anti-inflammatory effect of these compounds may be in link with Nrf2 acceleration as it well known that Nrf2 downregulates certain inflammatory pathways to maintain a balance. However, its intricate balance with NF-κB is critical in severe OS (Bhandari et al., 2021) Targeting NF-κB, a ubiquitous immunoregulator, with innovative therapies in SCH is an underexplored field that could help reduce symptoms in cases with active neuroinflammation (Murphy et al., 2021).

In terms of apoptosis, OS and elevated ROS produce 8-oxodG, which causes apoptosis in astrocytes, exposing them to neurodegeneration (Rasool et al., 2021). In the prefrontal cortex; Social isolation reinforced the apoptotic pathway which is accompanied by translocation of pro-apoptotic Bax to mitochondria and the anti-apoptotic Bcl-2 from the mitochondrial membrane to the cytoplasm and caspase-3 activation (Filipović et al., 2017).

Herein in SI model, enhanced apoptotic pathway appeared as increased BAX and Caspase-3 protein expression with decreased expression level of the anti-apoptotic protein Bcl-2 and this apoptotic cell death may in relation with excessive Oxidative stress (Kim et al., 2020; Rasool et al., 2021).

On the other hand, we found that Rutin has anti-apoptotic effect via decrease of BAX and Caspase-3 along with elevation of Bcl2 (Saafan et al., 2023). In the same context, Sodium selenite and Selenium nanoparticles biosynthesized using Rutin restore these alterations by decreasing BAX level and Caspase-3 activity and up-regulating Bcl2 (Yuan et al., 2020; Mohamed et al., 2023).

The dopamine system is involved in changes in social behaviours caused by persistent social isolation. This discovery offers light on the process underlying anomalies in social behaviour caused by chronic social isolation and suggests a promising target to treat mental illnesses related to social isolation (Zhang et al., 2021). Any abnormalities in GABA interneurons and inhibitory neurotransmission play a causative role in the onset of stress-related neurological disorder, and glycine works as a potent inhibitory neurotransmitter by attaching to its receptor (Ghosal et al., 2017; de Bartolomeis et al., 2020).

In the same setting, serotonin is essential for adapting behaviour to changing environmental demands. Cognitive flexibility is necessary for successful goal attainment and social relationships, and it is typically compromised in neuropsychiatric diseases (Luo et al., 2024).

MAOs are a class of neuronal enzymes (neuroenzymes) that play critical roles in neurodegeneration and serve as important biological targets in neuroscience. Thus, MAOs play a crucial function in the central and peripheral neurological systems by controlling the levels of monoamine neurotransmitters, as increasing MAO activity is related with loss of dopamine (Yeung et al., 2019).

Our findings support the fact that social isolation resulted in an abnormal neurochemical indicators manifested by decreased prefrontal dopamine, serotonin and GABA and elevated MAO and moreover caused neurological degeneration in the brain as indicated by a remarkable rise in brain AChE activity (Ma et al., 2017; Zhang et al., 2021). So collectively, it has been concluded that social isolation may exhibit mental conditions such as SI provoked SCH. It affects Neurotransmitters as dopamine, serotonin and GABA. So the pathophysiological impacts of social isolation include cholinergic system dysfunction, PS and inflammation (Mumtaz et al., 2018).

In co-treated groups, we found that the ameliorative role of Rutin against social isolation neurotoxicity indicated by a significant enhancement of dopamine, serotonin and GABA together with decreased MAO and AchE activities (Ahmed and Hussein, 2017; Oboh et al., 2019; Anesti et al., 2020).Moreover, Sodium selenite improved brain dysfunction induced by social isolation by enhancing dopamine and serotonin and GABA together with decreased MAO and AchE activities (Li et al., 2022). Notably, Selenium nanoparticles biosynthesized using rutin enhanced dopamine, serotonin and GABA with decreased MAO and AchE activities (Yuan et al., 2020; Mohamed et al., 2023).

Social isolation causes major behavioral and neurochemical dysfunctions in mice (Li et al., 2019). Previous research suggested that epigenetic modulation of BDNF in the prefrontal cortex could be one of the biological pathways that caused cognitive impairment (Li et al., 2016). Furthermore, the expression of the GFAP gene has received a lot of interest because it is a marker for astrocyte formation. GFAP upregulation is a hallmark for reactive gliosis, and its dominance in astrocytes presents a tool for their genetic manipulation (Brenner and Messing, 2021).

Depending on these data; we found that Social isolation decreased BDNF and increased GFPA gene expressions in the prefrontal cortex, this result in the same line with that noted by Sun et al. (2017a), Kim et al. (2020), Shirenova et al. (2021) suggesting neuronal damage.

In the same context, Rutin and Sodium selenite increased BDNF and decreased GFPA gene expressions (Çelik et al., 2020; Sreelatha et al., 2024).

Following the treatment of rats using Selenium nanoparticles biosynthesized using rutin; it produced high gene expression of BDNF and downregulated GFPA gene expression (Alrashdi et al., 2023; Mohamed et al., 2023).

This upregulation of BDNF may be occurred by TNF-α inhibition depending on the fact that there is a negative correlation between the BDNF and TNF-α level in the cases (Zhang et al., 2017).

Regarding cognitive impairment, rats subjected to social isolation exhibited behavioral alterations resemble those observed in SI. In the open field test, social isolation caused decrease in the number of line crossings, the rearing activity and sucrose preference. Also, the social interaction test showed that social isolation also resulted in severe social deficiencies in form of prolonged latency to initiate interaction, few contacts with one another and shortened the total interaction time and this result in the same line with that of Estaphan et al. (2021), Gamal El-Deen et al. (2025). These behavioural impairments may be linked to BDNF deficit, as new research reveals that disturbed signalling via BDNF may be implicated in mediating the deleterious impact of stress on the brain. SI in rats induces chronic stress and is extensively utilised as an animal model of mental diseases such as schizophrenia, supporting the possible involvement of BDNF in the aetiology of stress-related mental illness (Murínová et al., 2017).

In the opposite, co-administration of Rutin revealed remarkable ameliorations of SI like-symptoms in the majority of our behavioral tests. Rutin significantly improved the number of line crossings, the rearing activity and sucrose preference in the open field test. Also in the social interaction test, it restored the latency to initiate interaction, number of contacts ad interaction time.

Moreover, the animals co-treated with Sodium Selenite remained depression-free as denoted by their increased sucrose preference, rearing and line crossing in open field test and increased the interaction time and numbers in the social interaction test as mentioned by Bampi et al. (2019) and Rehman et al. (2022). Notably, our results showed that SeNPs reversed the depression-like behavior and cognitive impairment induced by social isolation; increased sucrose preference, line crossing and rearing activity in the open field test and also declined the latency to initial contact, prolonged interaction period and increase the number of contacts in social interaction tests (Albrakati et al., 2021; Gamal El-Deen et al., 2025).

This ameliorative effect may duo to their ability to dysregulate TNF-α as its elevation is associated with more rapid cognitive decline and has robust acute effects on brain function (Hennessy et al., 2017).

Regarding histopathological examination, it was parallel with the biochemical and behavioral outcomes. Our work revealed that social isolation caused aberrations in the prefrontal cortex architecture as described by Gamal El-Deen et al. (2025), these alterations may be in relation with the susceptibility of prefrontal cortex to oxidative, inflammation and apoptosis (Filipović et al., 2017). But In SeNPs group, the prefrontal cortical sections approximately normal appearance of cortical tissue with normal neurons and neuropil (Elfakharany et al., 2024).

The relationship between SCH and SI is critically important because it influences disease risk, clinical presentation, and long-term outcomes. SI is not only a common consequence of SCH, driven by negative symptoms, cognitive impairment, and stigma, but also a significant risk factor that may precede illness onset. Early and persistent social withdrawal has been associated with heightened stress vulnerability, dysregulated dopaminergic signaling, and impaired social cognition, all of which are central to SCH pathophysiology. Therefore, understanding and addressing SI is essential for improving early detection, therapeutic strategies, and quality of life in individuals with SCH, highlighting its relevance as both a target for intervention and a key modifier of disease progression.

Finally depending on all these results; nutritional therapies are required for people with SI who are receiving insufficient antioxidants, which increases oxidative stress and influences illness development. Therefore, proper nutrition, especially enough consumption of dietary antioxidants, should be advocated in people with SI (Karagöl et al., 2023). The study has limitation for including prefrontal cortex only, hippocampal tissue will be included in further studies.

## 5 Conclusion

Social isolation induced marked behavioral, neurochemical, inflammatory, and structural impairments consistent with SI. While olanzapine, free rutin, and sodium selenite each exerted partial protective effects, rutin-conjugated selenium nanoparticles demonstrated the most consistent and robust functional improvement across behavioral, biochemical, and histopathological parameters under the present experimental conditions. These findings suggest that nano-formulation may enhance the therapeutic performance of selenium–rutin systems; however, further studies are required to confirm nano-specific mechanistic and pharmacokinetic advantages.

## 6 Study limitations

Limitations of this study are related to the fact that there was no pharmacokinetic and biodistribution analysis to prove that nanoparticles have nano-specific benefits, as well as no physiological stability, elemental validation, and variability of batches analysis of nanoparticles, but only basal DLS, zeta potential, TEM, and FTIR analysis. Further, the molecular docking findings were only exploratory, and did not receive experimental confirmation, and thus can be deemed as mechanistic indications of target engagement, as opposed to concrete evidence of target engagement.

## Data Availability

The original contributions presented in the study are included in the article/supplementary material, further inquiries can be directed to the corresponding authors.
